# A proteomic survey of microtubule-associated proteins in a R402H TUBA1A mutant mouse

**DOI:** 10.1371/journal.pgen.1009104

**Published:** 2020-11-02

**Authors:** Ines Leca, Alexander William Phillips, Iris Hofer, Lukas Landler, Lyubov Ushakova, Thomas David Cushion, Gerhard Dürnberger, Karel Stejskal, Karl Mechtler, David Anthony Keays

**Affiliations:** 1 Research Institute of Molecular Pathology (IMP), Vienna Biocenter (VBC), Vienna, Austria; 2 Institute of Zoology, University of Natural Resources and Life Sciences (BOKU), Vienna, Austria; 3 Gregor Mendel Institute (GMI), Austrian Academy of Sciences, Vienna Biocenter (VBC), Vienna, Austria; 4 Institute of Molecular Biotechnology of the Austrian Academy of Sciences (IMBA), Vienna Biocenter (VBC), Vienna, Austria; 5 Department of Anatomy and Neuroscience, University of Melbourne, Parkville, Australia; 6 Division of Neurobiology, Department Biology II, Ludwig-Maximilians-University Munich, Planegg-Martinsried 82152, Germany; University of Colorado, UNITED STATES

## Abstract

Microtubules play a critical role in multiple aspects of neurodevelopment, including the generation, migration and differentiation of neurons. A recurrent mutation (R402H) in the α-tubulin gene *TUBA1A* is known to cause lissencephaly with cerebellar and striatal phenotypes. Previous work has shown that this mutation does not perturb the chaperone-mediated folding of tubulin heterodimers, which are able to assemble and incorporate into the microtubule lattice. To explore the molecular mechanisms that cause the disease state we generated a new conditional mouse line that recapitulates the R402H variant. We show that heterozygous mutants present with laminar phenotypes in the cortex and hippocampus, as well as a reduction in striatal size and cerebellar abnormalities. We demonstrate that homozygous expression of the R402H allele causes neuronal death and exacerbates a cell intrinsic defect in cortical neuronal migration. Microtubule sedimentation assays coupled with quantitative mass spectrometry demonstrated that the binding and/or levels of multiple microtubule associated proteins (MAPs) are perturbed by the R402H mutation including VAPB, REEP1, EZRIN, PRNP and DYNC1l1/2. Consistent with these data we show that the R402H mutation impairs dynein-mediated transport which is associated with a decoupling of the nucleus to the microtubule organising center. Our data support a model whereby the R402H variant is able to fold and incorporate into microtubules, but acts as a gain of function by perturbing the binding of MAPs.

## Author summary

Microtubules are polymers composed of tubulin proteins, which play an important role in the development of the human brain. Genetic mutations in tubulin genes are known to cause neurodevelopmental diseases, including lissencephaly which is characterised by an impairment in the migration of neurons. In this study we investigate how a common mutation (R402H) in *TUBA1A* causes lissencephaly by generating and characterising a mouse with the same variant. We show that affected animals recapitulate multiple aspects of the human disease; including laminar perturbations in the cortex and hippocampus, attributable to defects in neuronal migration at key developmental time points. To characterize the molecular implications of the R402H mutation we purified microtubules from the developing brain, and analysed the proteins present by mass spectrometry. This revealed that the binding of DYNC1I1/2 to microtubules is altered in mice with the R402H mutation. Our results provide insight into the molecular pathology underlying tubulin related disease states, and provide a foundation for the rational design of therapeutic interventions.

## Introduction

Consisting of α and β tubulin heterodimers, microtubules are dynamic polymers that play a critical role in multiple aspects of neuronal development, including the generation, migration and differentiation of neurons [1,2]. Neuronal migration in particular, relies on a dynamic cytoskeleton. As neurons exit from the proliferative ventricular zone they transition from a multipolar to a bipolar morphology, migrating along radial glia into the developing cortical plate [3]. This process requires an array of molecules that are responsible for a dramatic reorganisation of the cell’s cytoskeletal architecture, and the generation of force to translocate the nucleus [4]. Genetic studies in humans have identified some of the key molecular players that regulate cytoskeletal function during this process [2]. For example, doublecortin (DCX) is a microtubule-associated protein and LIS1 plays an important role in regulating dynein, a cytoskeletal molecular motor [5,6]. Mutations in DCX and LIS1 genes have been shown to cause lissencephaly, whilst dynein variants are associated with a range of cortical malformations [7–9].

Consistent with these reports, mutations in the α, β and γ tubulins, are known to cause a spectrum of neurodevelopmental disorders collectively referred to as tubulinopathies [10]. This includes lissencephaly (*TUBA1A*), polymicrogyria (*TUBA1A*, *TUBB2B*, *TUBB3*), microcephaly (*TUBB2B*, *TUBB3*, *TUBB5*, *TUBG1*), and oculomotor disorders (*TUBB2B*, *TUBB3*) [1,11–14]. The underlying genetic and molecular mechanisms that give rise to these different phenotypes are poorly understood. Some mutations appear to act as hypomorphs, as they profoundly affect the ability of tubulin heterodimers to fold, and it has been shown that null mutations in *Tubb5* recapitulate a microcephalic phenotype in mice [15,16]. Conversely, the ablation of *Tubb3* does not cause any overt neurodevelopmental phenotypes in rodents whereas a missense mutation (R262C) in this gene causes defects in the guidance of commissural axons and cranial nerves [17,18]. In addition to this, almost all tubulin mutations in patients are heterozygous *de novo* variants [19] and many of these missense variants have no effect on chaperone mediated tubulin folding. Taken together, this is indicative of a gain of function mechanism.

One mutation of particular interest is the R402H variant in *TUBA1A*, which has been reported in multiple unrelated patients with lissencephaly, whom also present with cerebellar hypoplasia and striatal abnormalities [20–23]. The R402 residue is located close to the C-terminus of α-tubulin, at the beginning of the H11-H12 loop, and faces the surface of microtubules [24]. We have previously shown that the R402H mutation does not perturb the chaperone-mediated folding of tubulin heterodimers, and that R402H mutant heterodimers can integrate into the interphase cytoskeleton [16]. These findings have led to the hypothesis that the R402H mutation acts by a gain of function mechanism perturbing the binding of microtubule associated proteins (MAPs). This concept has been supported by *in utero* overexpression of the R402H mutation in mice, and the generation of transgenic yeast harbouring an analogous variant (R403H) [25]. Here, we explore this idea further by exploiting a new R402H conditional *TUBA1A* mouse mutant. We show that this mouse line recapitulates multiple aspects of the human disease including neuronal migration defects in the cortex and hippocampus as well as a reduction in striatal and cerebellar size. Coupling microtubule sedimentation assays with quantitative mass spectrometry we identify a number of MAPs that are dysregulated in this mouse mutant including; VAPB, REEP1, EZRIN, PRNP and DYNC1l1/2.

## Results

### Generation of the *R402H Tuba1a* mouse line

**Fig 1:**
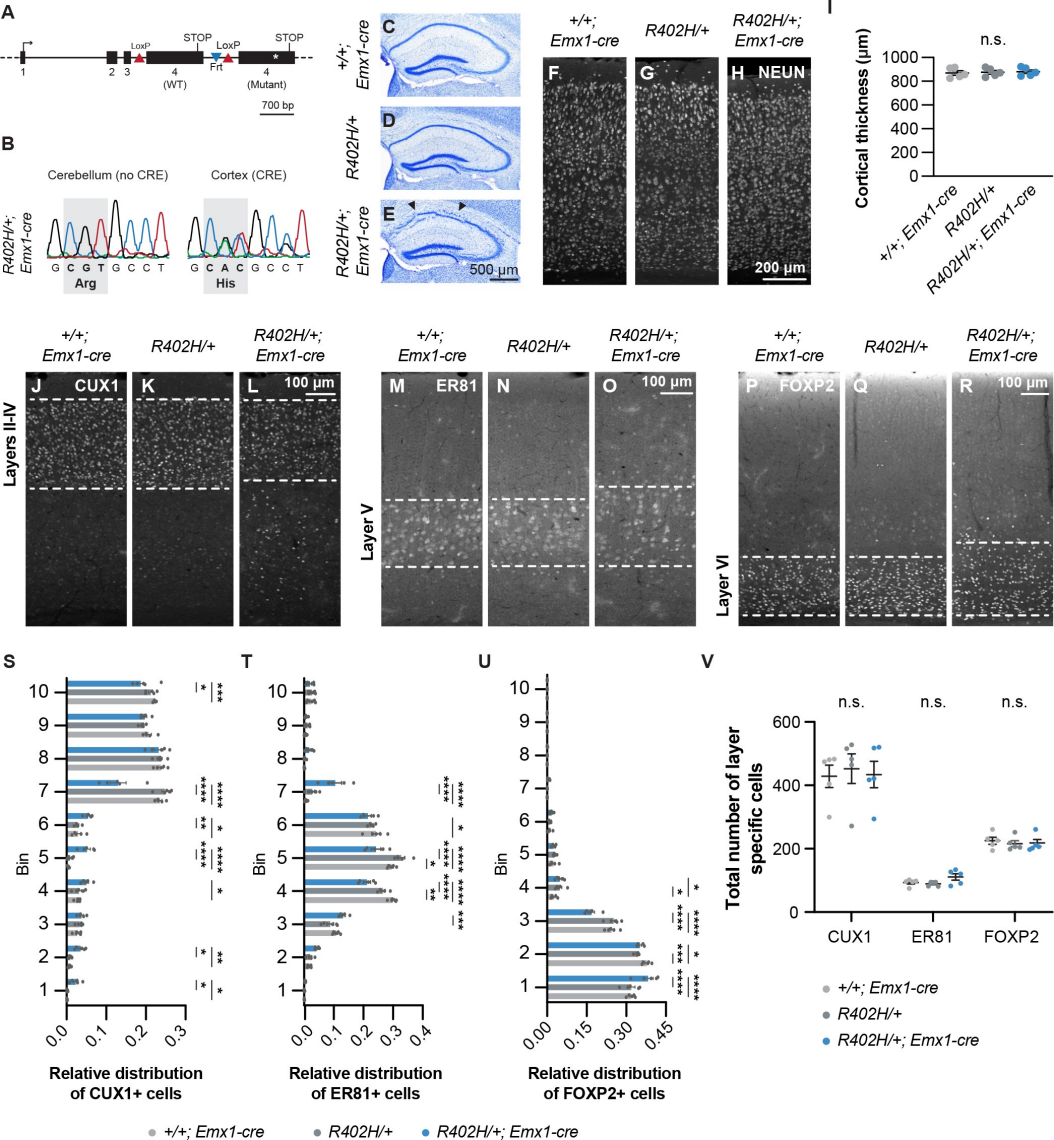
Laminar abnormalities in the hippocampus and cortex of *R402H/+; Emx1-cre* mice. (A) Schematic showing the targeting construct employed to generate *R402H Tuba1a* mice. The endogenous locus was replaced with two copies of exon 4, one flanked by LoxP sites and the other carrying the R402H mutation. In the presence of a Cre recombinase, the wild-type variant of exon 4 is excised, and the R402H mutant is expressed. (B) Sequencing traces generated from mRNA extracted from cortex and cerebellum of heterozygous *R402H/+* animals that express Emx1-Cre in the dorsal telencephalon, but not in the cerebellum. The R402H mutation is only detected in the cortex. (C-E) Nissl stained sections of adult *+/+; Emx1-cre*, *R402H/+*, and *R402H/+; Emx1-cre* animals. A fractured pyramidal cell layer in CA1 region (indicated by arrowheads) of the hippocampus is present in *R402H/+; Emx1-cre* animals (n = 5). (F-H) Representative coronal sections of the adult cortex stained with the pan-neuronal marker NEUN from *+/+; Emx1-cre*, *R402H/+*, and *R402H/+; Emx1-cre* animals. (I) Quantification of cortical thickness reveals no significant difference between genotypes (n = 5, P>0.05). (J-R) Cortical sections stained with layer-specific markers: CUX1 for layers II to IV, ER81 for layer V and FOXP2 for layer VI. (S-U) Relative distribution of CUX1 (S), ER81 (T) and FOXP2 (U) positive cells in control mice (*+/+; Emx1-cre* in light grey and *R402H/+* in dark grey) and heterozygous mutants (*R402H/+; Emx1-cre* in blue). *R402H/+; Emx1-cre* mutants presented with significant lamination defects for all three layer-markers (n = 5, see S1 Table). (V) The number of CUX1, ER81 and FOXP2 positive neurons in the somatosensory cortex. No significant difference (n = 5, P>0.05) was observed when comparing controls and *R402H/+; Emx1-cre* mutants. Error bars show mean ± s.e.m. *P<0.05; **P<0.01; ***P< 0.001; ****P< 0.0001. Scale bars show 700 bp in A, 500 μm in E, 200 μm in H and 100 μm in L, O and R.

To generate a conditional *R402H Tuba1a* mouse mutant we designed a targeting construct that carries two copies of the terminal exon 4 (Fig 1A, S1A and S1B Fig). The first copy encodes wild-type *Tuba1a* and is flanked by LoxP sites. Downstream we cloned a R402H mutant version of exon 4, that is expressed in the presence of a Cre recombinase. Following the targeting of ES cells, southern blot analysis, and blastocyst injection, chimeric offspring were backcrossed to C57/BL6. We confirmed the functionality of our genetic approach by crossing these animals with a Emx1-Cre driver line that expresses Cre in the dorsal forebrain [26]. cDNA sequencing of *R402H/+; Emx1-cre* animals showed that the transcript encoding the R402H mutation is expressed in the cortex of adult mice, but not in the cerebellum (Fig 1B). We confirmed that our manipulation of the *Tuba1a* genomic locus by itself does not lead to any overt phenotypes by assessing the neuroanatomical features of heterozygous and homozygous adult animals that do not express Cre recombinase. Both *R402H/+* and *R402H/R402H* are indistinguishable from *+/+* controls (S1C–S1E and S1F–S1I Fig).

### Laminar abnormalities in the hippocampus and cortex of *R402H/+; Emx1-cre* mice

We proceeded by analysing the brain morphology of *R402H/+; Emx1-cre* mice by Nissl staining. This revealed a fractured pyramidal cell layer in the CA1 region of the hippocampus with ectopic cells in the stratum oriens reminiscent of the *S140G TUBA1A* mutant (n = 5, Fig 1C–1E) [20]. We stained adult brains (n = 5) with the pan-neuronal marker NeuN, and quantified cortical thickness. No significant differences were observed when comparing *R402H/+; Emx1-cre* mice and wild-type littermate controls (Fig 1F–1I) (n = 5, *R402H/+* vs. *R402H/+; Emx1-cre*, P>0.05). To assess whether the laminar structure of the cortex was affected in *R402H/+; Emx1-cre* animals we stained adult sections with antibodies that label specific populations of neurons expressing CUX1 (layers II-IV, Fig 1J–1L), ER81 (layer V, Fig 1M–1O) and FOXP2 (layer VI, Fig 1P–1R) [27]. CUX1^+^ neurons were scattered across the deep layers of the cortex and both layers V and VI appeared less defined in *R402H/+; Emx1-cre* mutants, with a reduction in the density of labelled cells. Blind quantification showed that the distribution of CUX1, ER81 and FOXP2 positive cells in *R402H/+; Emx1-cre* mutants was significantly different from controls (n = 5, S1 Table) (Fig 1S–1U), but with no difference in total cell numbers (Fig 1V) (n = 5, *R402H/+* vs. *R402H/+; Emx1-cre*, P>0.05). These results show that the R402H mutation causes abnormalities in both the cortex and hippocampus in mice.

### Defects in neuronal migration in the cortex and hippocampus in *R402H/+; Emx1-cre* mice

**Fig 2:**
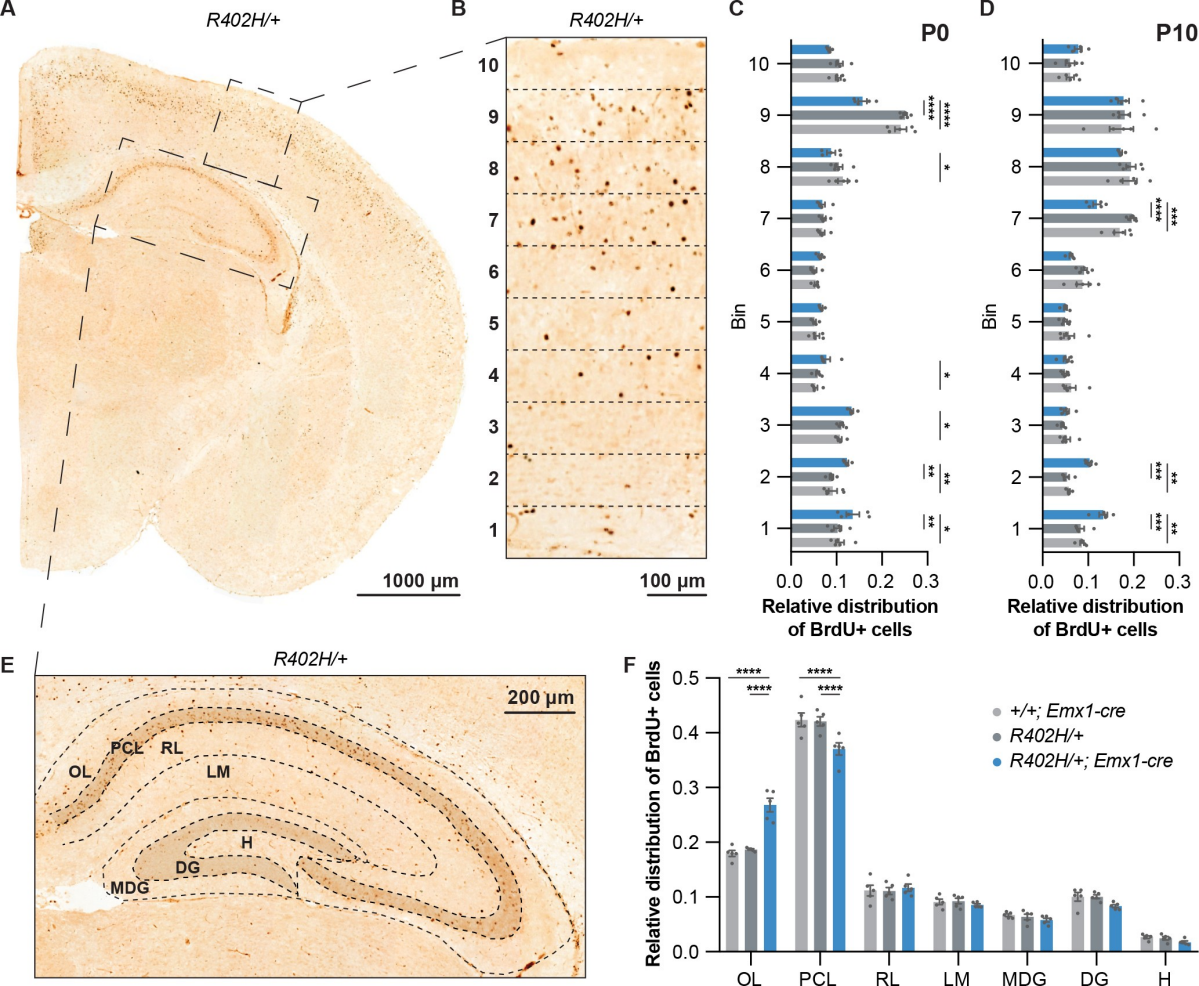
Neuronal migration defects in the cortex and hippocampus of *R402H/+; Emx1-cre* mice. BrdU was injected into pregnant dams at E14.5, and brains harvested at either P0 (n = 5) or P10 (n = 5). (A) Image of a P10 brain stained with antibodies that target BrdU. (B) Enlargement showing segmentation of the P10 cortex into 10 bins. (C) Graph showing the relative distribution of BrdU+ cells in the cortex of P0 control animals (*+/+; Emx1-cre* in light grey and *R402H/+* in dark grey) and heterozygous mutants (*R402H/+; Emx1-cre* in blue). In *R402H/+; Emx1-cre* animals we noted a significant accumulation of BrdU+ cells in the deeper layers of the cortex (bins 1–4) concomitant with a decrease of BrdU+ cells in the upper layers (bins 8–9). (D) Relative distribution of BrdU+ cells across the cortex at P10. We found a significant increase in the proportion of BrdU+ cells in the deeper layers (bins 1–2) and a reduction in proportion of BrdU+ cells in bin 7. (E) Image showing the segmentation of a P10 hippocampus stained with antibodies that target BrdU. We quantitated BrdU+ cells in the oriens layer (OL), pyramidal cell layer (PCL), radiatum layer (RL), lacunosum moleculare (LM), molecular dentate gyrus (MDG), dentate gyrus (DG) and the hilus (H). (F) Graph showing the distribution of BrdU+ cells in the different layers of the hippocampus of P10 mice (n = 5). *R402H/+; Emx1-cre* mutants show a significant accumulation of cells in the OL, accompanied by a decrease in BrdU+ cells in the PCL in comparison to controls. Error bars show mean ± s.e.m. *P<0.05; **P<0.01; ***P< 0.001; ****P< 0.0001 (See also S1 Table). Scale bars show 1000 μm in A, 100 μm in B and 200 μm in E.

To directly assess neuronal migration in *R402H/+; Emx1-cre* mice, we injected pregnant dams with BrdU at E14.5 and harvested brains at P0 and P10. We divided the cortex into 10 bins and quantitated the distribution of BrdU-positive cells in *R402H/+; Emx1-cre* mutants and littermate controls (Fig 2A–2D). At P0 we observed a significant reduction in the relative number of BrdU positive cells in bin 9 in *R402H/+; Emx1-cre* mutants with a concomitant increase in bins 1–2 (Fig 2C) (n = 5, *R402H/+* vs. *R402H/+; Emx1-cre* bin 9: P<0.0001, bin 1: P<0.01 and bin 2: P<0.01). At P10 there was a significant reduction of cells in bin 7, with an accumulation of BrdU-positive cells in bins 1 and 2 in *R402H/+; Emx1-cre* mutants, when compared to their littermate controls (Fig 2D) (n = 5, *R402H/+* vs. *R402H/+ Emx1-cre*, bin 7: P<0.0001, bin 1: P<0.001 and bin 2: P<0.001). To assess neuronal migration in the hippocampus we focused on our P10 cohort and quantitated BrdU-positive cells in the oriens layer, the pyramidal cell layer, the radiatum layer, the lacunosum moleculare, the molecular layer of the dentate gyrus, the dentate gyrus and in the hilus (Fig 2E and 2F). This showed a significant increase in the relative number of BrdU-positive cells in the oriens layer in *R402H/+; Emx1-cre* mutants in comparison to littermate controls, with a decrease in the pyramidal cell layer (n = 5, *R402H/+* vs. *R402H/+; Emx1-cre* oriens layer: P<0.0001, pyramidal cell layer: P<0.0001). Quantitation of the total number of BrdU-labelled neurons in both the cortex and hippocampus revealed no differences between genotypes suggesting that neurogenesis is normal in *R402H/+; Emx1-cre* animals (S2 Fig) (n = 5, *R402H/+* vs. *R402H/+; Emx1-cre*, P>0.05). These data show that the R402H mutation in TUBA1A causes defects in neuronal migration in the cortex and hippocampus of mice.

### Reduction in striatal size and cerebellar abnormalities in *R402H/+; Nestin-cre* mice

**Fig 3:**
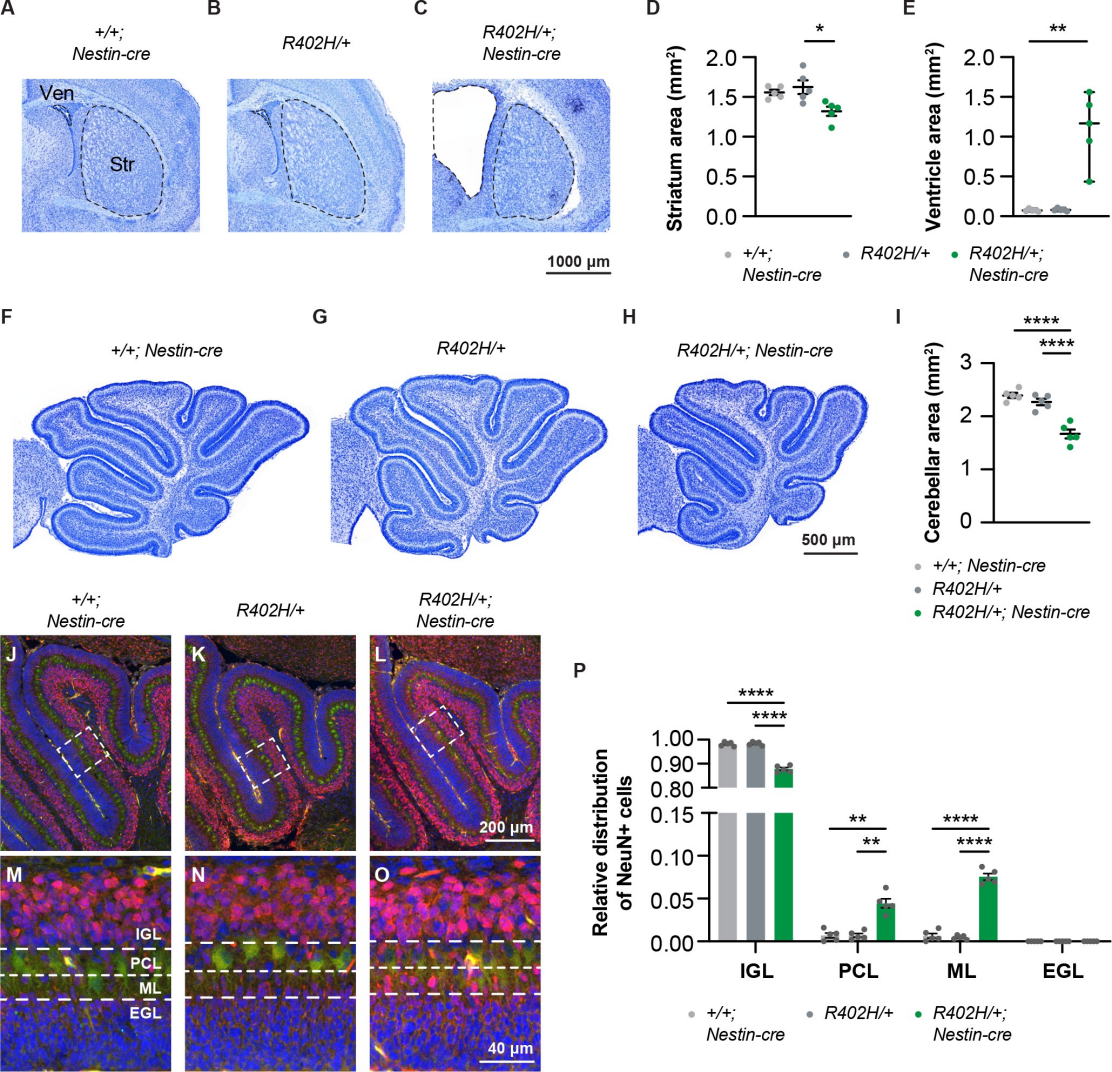
Reduction in striatal size and cerebellar abnormalities in *R402H/+; Nestin-cre* mice. *R402H/+* animals were crossed with a pan neuronal Nestin-cre driver line. (A-C) Representative coronal brain sections of *+/+; Nestin-cre* controls, *R402H/+* controls, and *R402H/+; Nestin-cre* mutants at post-natal day 6 (P6) stained with Nissl. Dashed lines indicate the area measured for the ventricles (Ven) and striatum (Str). (D-E) Quantification revealed a significant difference in the striatal (D) and ventricular area (E) when comparing *+/+; Nestin-cre* controls, *R402H/+* controls, and *R402H/+; Nestin-cre* mutants (n = 5, striatum: *R402H/+* vs. *R402H/+; Nestin-cre* P<0.05, ventricle: *+/+; Nestin-cre* vs. *R402H/+; Nestin-cre* P<0.01). (F-H) Representative sagittal sections of the cerebellum at P6. (I) *R402H/+; Nestin-cre* mutants show a significant reduction of the cerebellar area compared to controls (n = 5, *R402H/+* vs. *R402H/+; Nestin-cre* P<0.0001). (J-L) Representative cerebellar sagittal sections at P6 in which Purkinje cells were labeled with Calbindin (green) and neurons with NEUN (red). Sections were also counterstained with DAPI (in blue). (M-O) Enlargements of the boxed areas shown in (J-L). The internal granular layer (IGL), Purkinje cell layer (PCL), molecular layer (ML) and external granular layer (EGL) are marked. (P) Relative distribution of NEUN-positive neurons in the four layers of the cerebellum. There is a significant reduction in NEUN-positive cells found in the IGL and a significant increase in both the PCL and ML, in *R402H/+; Nestin-cre* mutants compared to littermate controls (n = 5, IGL: *R402H/+* vs. *R402H/+; Nestin-cre* P<0.0001, PCL: *R402H/+* vs. *R402H/+; Nestin-cre* P<0.01 and ML: *R402H/+* vs. *R402H/+; Nestin-cre* P<0.0001). In all panels *+/+; Nestin-cre* controls are shown in light grey, *R402H/+* controls in dark grey and *R402H/+; Nestin-cre* mutants in green. Error bars show the mean ± s.e.m. except for E, where data is represented as median ± 95% CI. *P<0.05, **P<0.01, ****P< 0.0001. Scale bars show 1000 μm in C, 500 μm in H, 200 μm in L and 40 μm in O.

A recurrent feature described in patients with TUBA1A mutations is a dysmorphic striatum with a hypoplastic and/or dysplastic cerebellum [28,29]. To assess whether the *R402H/+* mouse line recapitulates these features we exploited a Nestin driver line that expresses Cre recombinase throughout the developing nervous system [30]. To determine whether the striatum was affected in the mutants, we stained coronal brain sections of P6 mice with Nissl (Fig 3A–3C). There was a significant reduction in the striatal area in *R402H/+; Nestin-cre* animals in comparison to littermate controls (Fig 3D) (n = 5, *R402H/+* vs. *R402H/+; Nestin-cre* P<0.05), accompanied by an enlargement of the lateral ventricles (Fig 3E) (n = 5, *+/+; Nestin-cre* vs. *R402H/+; Nestin-cre* P<0.01). This phenotype was mirrored in the cerebellum where we again observed a significant reduction in the total area of sagittal sections in P6 *R402H/+; Nestin-cre* animals (Fig 3F–3I) (n = 5, *R402H/+* vs. *R402H/+; Nestin-cre* P<0.0001). To assess cerebellar lamination, we focused on the 3^rd^ folia staining sagittal sections with calbindin (which labels Purkinje cells) and NeuN (which labels all neurons) (Fig 3J–3O). We quantitated the number of NeuN-positive cells within the internal granule cell layer (IGL), the Purkinje cell layer (PCL), the molecular layer (ML) and in the external granule cell layer (EGL). We observed a significant accumulation of NeuN-positive cells in both the ML and PCL in *R402H/+; Nestin-cre* mutants, concomitant with a reduction in the number of NeuN-positive cells in the IGL (Fig 3P) (n = 5, *R402H/+* vs. *R402H/+ Nestin-cre* ML: P<0.0001, PCL: P<0.01, and IGL: P<0.0001). These results show that the R402H mutation causes a reduction in the size of the striatum and cerebellum, and defects in cerebellar lamination.

### Homozygous expression of the R402H mutation exacerbates the migration phenotype and leads to neuronal cell death

**Fig 4:**
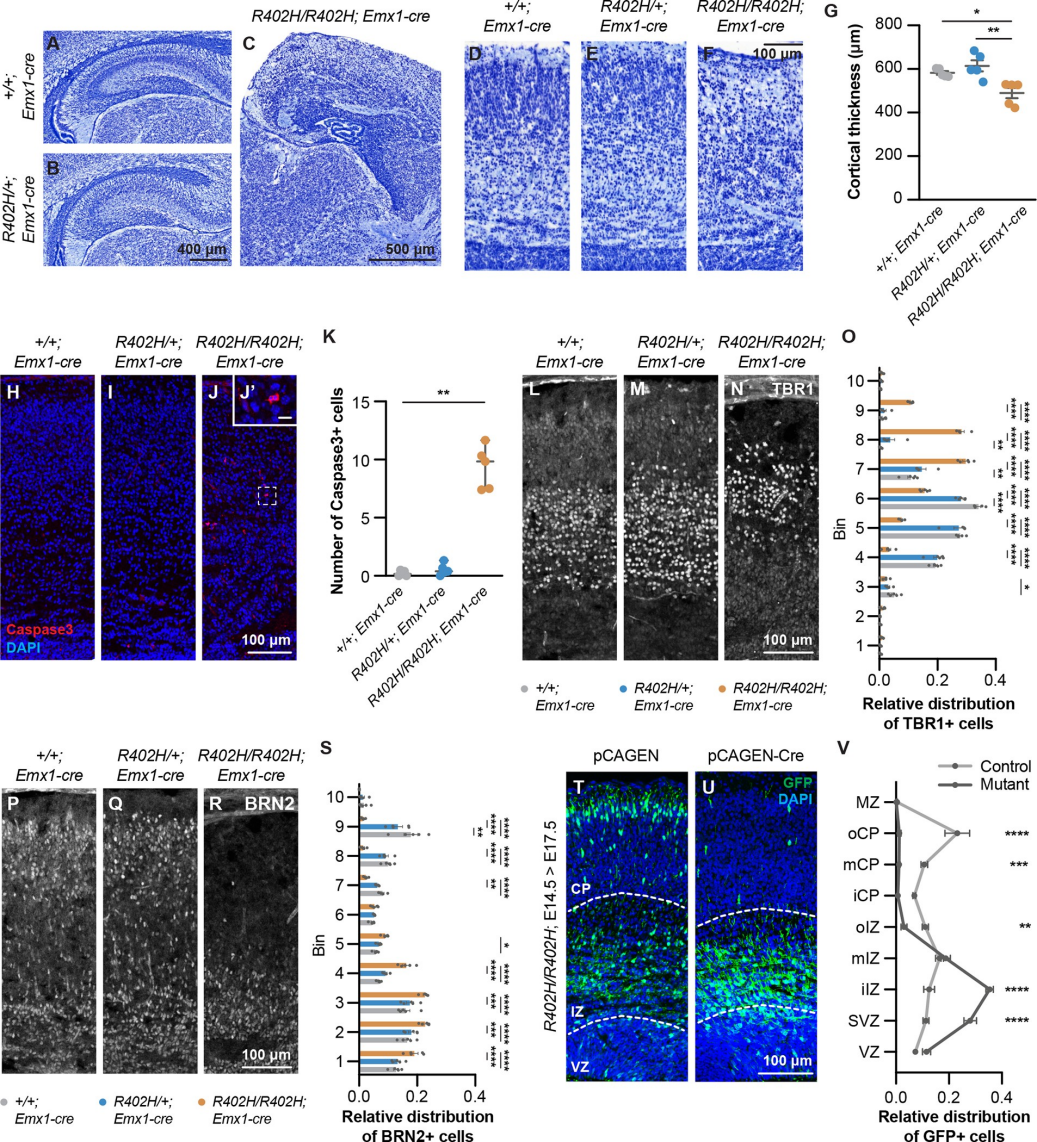
Homozygous expression of the R402H mutation exacerbates the migration phenotype and leads to neuronal cell death. (A-C) Nissl stained coronal sections from E18.5 embryos for (A) *+/+; Emx1-cre* controls, (B) *R402H/+; Emx1-cre* heterozygotes, and (C) *R402H/R402H; Emx1-cre* homozygous mutants. *R402H/R402H; Emx1-cre* mutants present with a severely dysmorphic hippocampus. (D-F) Nissl stained cortical sections from E18.5 embryos for (D) *+/+; Emx1-cre* controls, (E) *R402H/+; Emx1-cre* heterozygotes, and (F) *R402H/R402H; Emx1-cre* homozygous mutants. (G) Quantification of cortical thickness shows that *R402H/R402H; Emx1-cre* mutants exhibit a significant decrease in cortical thickness compared to littermates (n = 5, *+/+; Emx1-cre* vs. *R402H/R402H; Emx1-cre* P<0.05, *R402H/+; Emx1-cre* vs. *R402H/R402H; Emx1-cre* P<0.01). (H-J) Representative E18.5 sections stained with cleaved caspase-3. (K) Quantification of caspase-3-positive cells reveals a significant increase in *R402H/R402H; Emx1-cre* mutants compared to littermates (n = 5, *+/+; Emx1-cre* vs. *R402H/R402H; Emx1-cre* P<0.01). (L-N) Representative E18.5 sections stained with an antibody that targets TBR1 (which labels deep layer neurons) and (P-R) an antibody against BRN2 (which labels upper layer late-born neurons). (O, S) Quantifications showing the relative distribution of TBR1+ and BRN2+ cells across the developing cortex. In *R402H/R402H; Emx1-cre* homozygous mutants there was a cortical inversion, with BRN2+ neurons accumulating in the intermediate zone, and TBR1+ neurons present in the superficial layers (n = 5, see S1 Table). (T-V) *R402H/R402H* animals were electroporated with either a pCAGEN control vector or pCAGEN-Cre at E14.5 and the embryos collected at E17.5. (V) Relative distribution of GFP+ cells across the cortex at E17.5. Acute expression of the R402H allele results in an accumulation of neurons in the SVZ and iIZ, which is accompanied by a significantly lower proportion of neurons that reach the cortical plate (n = 6, see S1 Table). In all panels control animals (*+/+; Emx1-cre*) are shown in grey, heterozygotes (*R402H/+; Emx1-cre*) in blue and homozygotes (*R402H/R402H; Emx1-cre*) in orange. VZ denotes ventricular zone; SVZ, subventricular zone; IZ, intermediate zone; CP, cortical plate; MZ, marginal zone; iIZ, mIZ, oIZ, and iCP, mCP, oCP correspond to inner, medial and outer IZ and CP. Scale bars show 400 μm in B, 500 μm in C, 100 μm in F, 100 μm in J, N, R and U, and 10 μm in J’. In G, O, S and V error bars show mean ± s.e.m., and in K, data is represented as median ± 95% CI. *P<0.05; **P<0.01, ***P<0.001, ****P< 0.0001.

The conditional approach we employed allowed us to generate homozygous, *R402H/R402H; Emx1-cre* animals, however, these mice die shortly after birth. We therefore harvested *R402H/R402H; Emx1-cre* embryos at E18.5 when *Tuba1a* is expressed at high levels and neuronal migration is ongoing [31]. Nissl staining revealed a malformed hippocampus with no discernible pyramidal cell layer in *R402H/R402H; Emx1-cre* homozygotes (Fig 4A–4C), and a significant reduction in cortical thickness in comparison to *R402H/+; Emx1-cre* heterozygotes and *+/+; Emx1-cre* controls (Fig 4D–4G) (n = 5, *+/+; Emx1-cre* vs. *R402H/R402H; Emx1-cre* P<0.05, and *R402H/+; Emx1-cre* vs. *R402H/R402H; Emx1-cre* P<0.01). To ascertain if this reduction in cortical thickness is due to an increase in cell death, we stained sections with an antibody against cleaved caspase-3. Blind quantitation revealed a significant increase in caspase-3-positive cells in *R402H/R402H; Emx1-cre* homozygotes in comparison to *+/+; Emx1-cre* controls (Fig 4H–4K) (n = 5, *+/+ Emx1-cre* vs. *R402H/R402H; Emx1-cre*, P<0.01). To assess the organization of the cortex we stained E18.5 sections with antibodies that target TBR1 (which labels deep layer neurons) and BRN2 (which labels neurons in the superficial layers) and analysed their distribution in 10 equally sized bins that spanned the cortical plate (Fig 4L–4S) [27]. In *R402H/R402H; Emx1-cre* homozygotes we observed a cortical inversion, with the majority of TBR1-positive neurons in bins 7–10, and a higher percentage of BRN2-positive neurons in bins 1–4 (n = 5, S1 Table). Quantification confirmed a significant reduction in the total number of TBR1 and BRN2 neurons (S3 Fig). Next, we asked whether the abnormal positioning of neurons in *R402H/R402H; Emx1-cre* homozygotes is due to a cell intrinsic defect in neuronal migration. To address this question, we performed *in utero* electroporation employing a pCAGEN-Cre vector on *R402H/R402H* mice at E14.5 and collected embryos at E17.5 (Fig 4T–4U). As a control we performed electroporations with an empty pCAGEN vector. We quantified the percentage of GFP-positive cells in the ventricular zone (VZ), the subventricular zone (SVZ), the inner, medial, and outer intermediate zone (iIZ, mIZ, oIZ), and the inner, medial, and outer cortical plate (iCP, mCP, oCP). Quantification showed that Cre-expressing neurons arrest in the SVZ and IZ, failing to migrate into the cortical plate (Fig 4V) (n = 6, *R402H/R402H;* pCAGEN vs. *R402H/R402H;* pCAGEN-Cre SVZ P<0.0001, iIZ P<0.0001, oIZ P<0.01, mCP P<0.001, oCP P<0.0001). Taken together these data show that homozygous expression of the R402H allele causes neuronal death and a severe cell-intrinsic defect in neuronal migration.

### The R402H mutation alters the microtubule proteome

**Fig 5:**
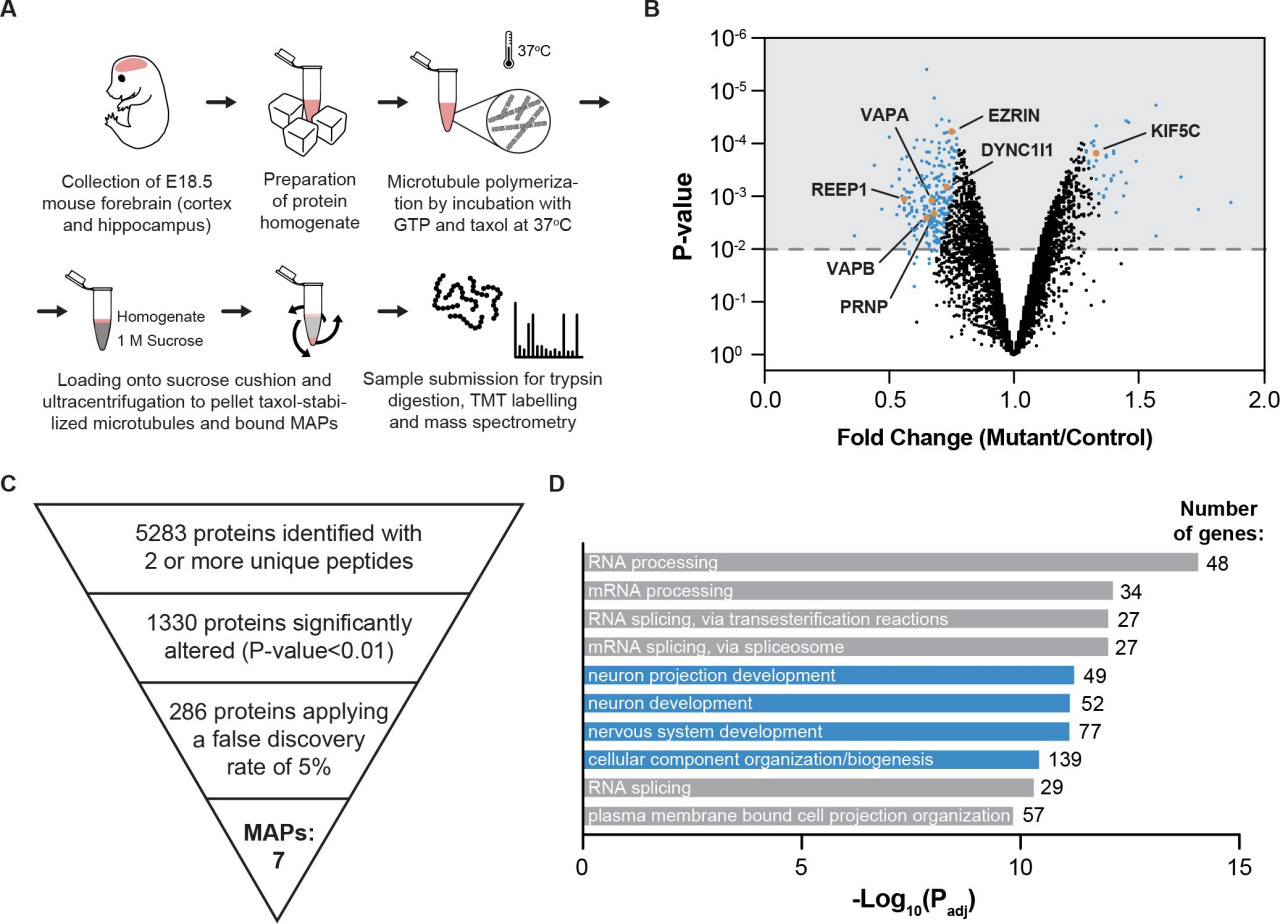
The R402H mutation alters the microtubule proteome. (A) Graphical depiction of the protocol employed to isolate microtubules and microtubule-associated proteins (MAPs) from the forebrain of E18.5 *R402H/R402H*; *Emx1-cre* mutants and *R402H/R402H* littermate controls (n = 4). (B) Volcano plot showing the results of our mass spectrometry analysis. In total we identified 5283 proteins, of which 1330 were significantly altered in *R402H/R402H*; *Emx1-cre* mutants (P<0.01) (shown in grey). These were narrowed to 286 proteins upon application of a false discovery rate of 5% (shown in blue). (C) Pyramid showing how we filtered our mass spectrometry dataset, resulting in the identification of seven known MAPs highlighted in orange in (B). (D) Gene ontology analysis performed with g:Profiler on the 286 proteins revealing enrichment for GO terms associated with cellular organization and nervous system development (highlighted in blue).

**Fig 6:**
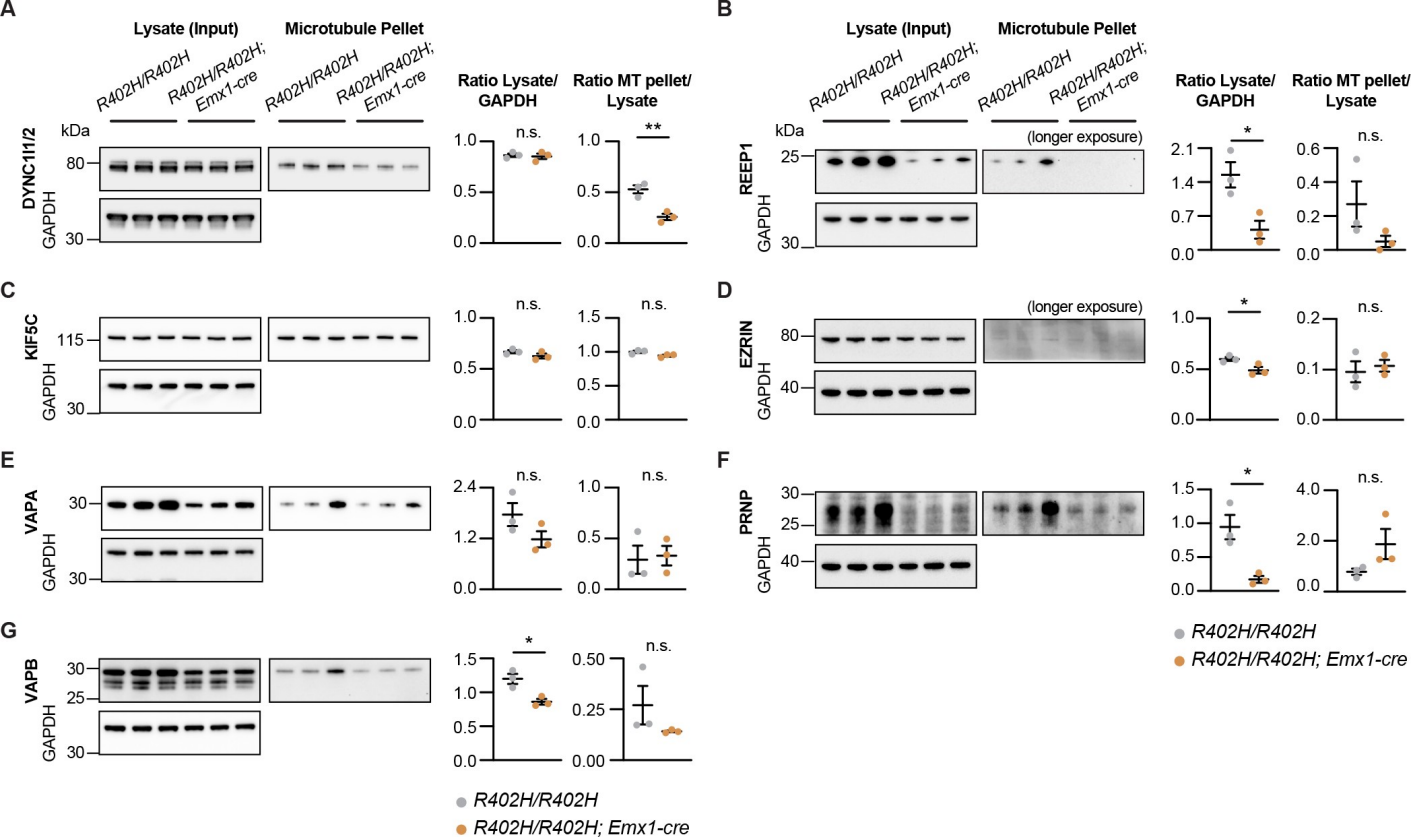
VAPB, REEP1, EZRIN, PRNP and DYNC1l1/2 are dysregulated in *R402H/R402H; Emx1-cre* mice. (A-G) Western blot analysis and quantification of the seven MAPs identified by mass spectrometry: (A) DYNC1l1/2; (B) REEP1; (C) KIF5C; (D) EZRIN; (E) VAPA; (F) PRNP; (G) VAPB. We quantified the total protein levels in brain lysates (normalised to GAPDH) and the protein present in microtubule pellets (normalised to lysate levels). Total levels of VAPB, REEP1, EZRIN and PRNP were significantly lower in the mutant lysates (n = 3, *R402H/R402H* vs. *R402H/R402H; Emx1-cre*, VAPB: P<0.05, REEP1: P<0.05, EZRIN: P<0.05, and PRNP: P<0.05). For DYNC1l1/2 total protein levels were comparable (n = 3, *R402H/R402H* vs. *R402H/R402H; Emx1-cre* P>0.05), but there was a significant reduction in DYNC1l1/2 levels that sedimented with microtubules in *R402H/R402H* mutants (n = 3, *R402H/R402H* vs. *R402H/R402H; Emx1-cre* P<0.01). Please note that GAPDH loading controls are the same for EZRIN (D) and PRNP (F); as the membrane was cut and probed with antibodies that target different sized proteins. Error bars show mean ± s.e.m. in all panels. Control animals (*R402H/R402H*) are represented in grey and homozygous mutants (*R402H/R402H; Emx1-cre*) in orange. *P<0.05 and **P<0.01. All immunoblots shown are cropped images; for full-length blots see S5 Fig.

To gain insight into the molecular mechanisms that underlie the neuronal migration phenotype in *R402H/R402H*; *Emx1-cre* animals, we isolated microtubules from the developing forebrain of mutants and littermate controls at E18.5 by taxol-mediated polymerisation coupled with sucrose gradient centrifugation (n = 4) (Fig 5A). We confirmed that expression of R402H TUBA1A did not affect α-tubulin protein levels in either brain lysates or microtubule pellets (S4A–S4C Fig) (n = 3, *R402H/R402H* vs. *R402H/R402H; Emx1-cre* P>0.05). Exploiting isobaric labelling and mass spectrometry, we undertook a quantitative comparison of the proteins associated with microtubules in *R402H/R402H*; *Emx1-cre* mutants and *R402H/R402H* controls. In total this experiment resulted in the identification of 5283 proteins with 2 or more unique peptides, including DCX, Lis1, multiple kinesin motors, as well as EB1, EB2 and EB3 (S2 Table). Amongst these 1330 were significantly altered in *R402H/R402H*; *Emx1-cre* mutants (P<0.01) (S3 Table). This number was narrowed to 286 following the application of a false discovery rate of 5% (Fig 5B and 5C, S4 Table). To assess the reliability of our data, we compared the results of four biological replicates and observed similar fold change ratios for those proteins altered in *R402H/R402H*; *Emx1-cre* mutants (S4D Fig). Next, we performed an unbiased gene ontology analysis on the 286 proteins altered in *R402H/R402H*; *Emx1-cre* mutants which revealed an enrichment of proteins involved in cellular component organization, neuron and neuron projection development (Fig 5D). We further refined our list of candidates to 7 proteins known to directly bind to microtubules; VAPA, VAPB, REEP1, EZRIN, PRNP, KIF5C and DYNC1l1. To confirm that these proteins are dysregulated in *R402H/R402H*; *Emx1-cre* mutants, we again isolated microtubules from the forebrain of E18.5 animals and undertook western blot analyses (Fig 6A–6G and S5 Fig). For each of our candidates we quantitated the total amount of protein in forebrain lysates, as well as the amount that sedimented with microtubules. Unexpectedly we found that the total amount of VAPB, REEP1, EZRIN and PRNP was significantly lower in the *R402H/R402H*; *Emx1-cre* brain lysates with a corresponding reduction in protein levels associated with microtubules (Fig 6B, 6D and 6F–6G) (n = 3, *R402H/R402H* vs. *R402H/R402H; Emx1-cre* VAPB: P<0.05, REEP1: P<0.05, EZRIN: P<0.05 and PRNP: P<0.05). We quantitated the mRNA levels of *Vapb*, *Reep1*, *Ezrin* and *Prnp* in E18.5 cortices in *R402H/R402H* controls and *R402H/R402H; Emx1-cre* mutants, but observed no significant differences in transcript levels (S6 Fig) (n = 5, *R402H/R402H* vs. *R402H/R402H; Emx1-cre*: VAPB: P>0.5, REEP1: P>0.5, EZRIN: P>0.1 and PRNP: P>0.1). These data suggest that the decreased protein observed in lysates is due to post-translation dysfunction, and not a transcriptional phenotype. Only in the case of dynein intermediate chain (DYNC1l1/2) were there comparable amounts of protein in control brain lysates but a significant reduction in protein levels that sedimented with microtubules in *R402H/R402H*; *Emx1-cre* mutants (Fig 6A) (n = 3, *R402H/R402H* vs. *R402H/R402H; Emx1-cre* lysate/GAPDH: P>0.05 and MT pellet/lysate: P<0.01).

### The R402H mutation perturbs dynein-mediated lysosomal transport and coupling of the nucleus to the centrosome

**Fig 7:**
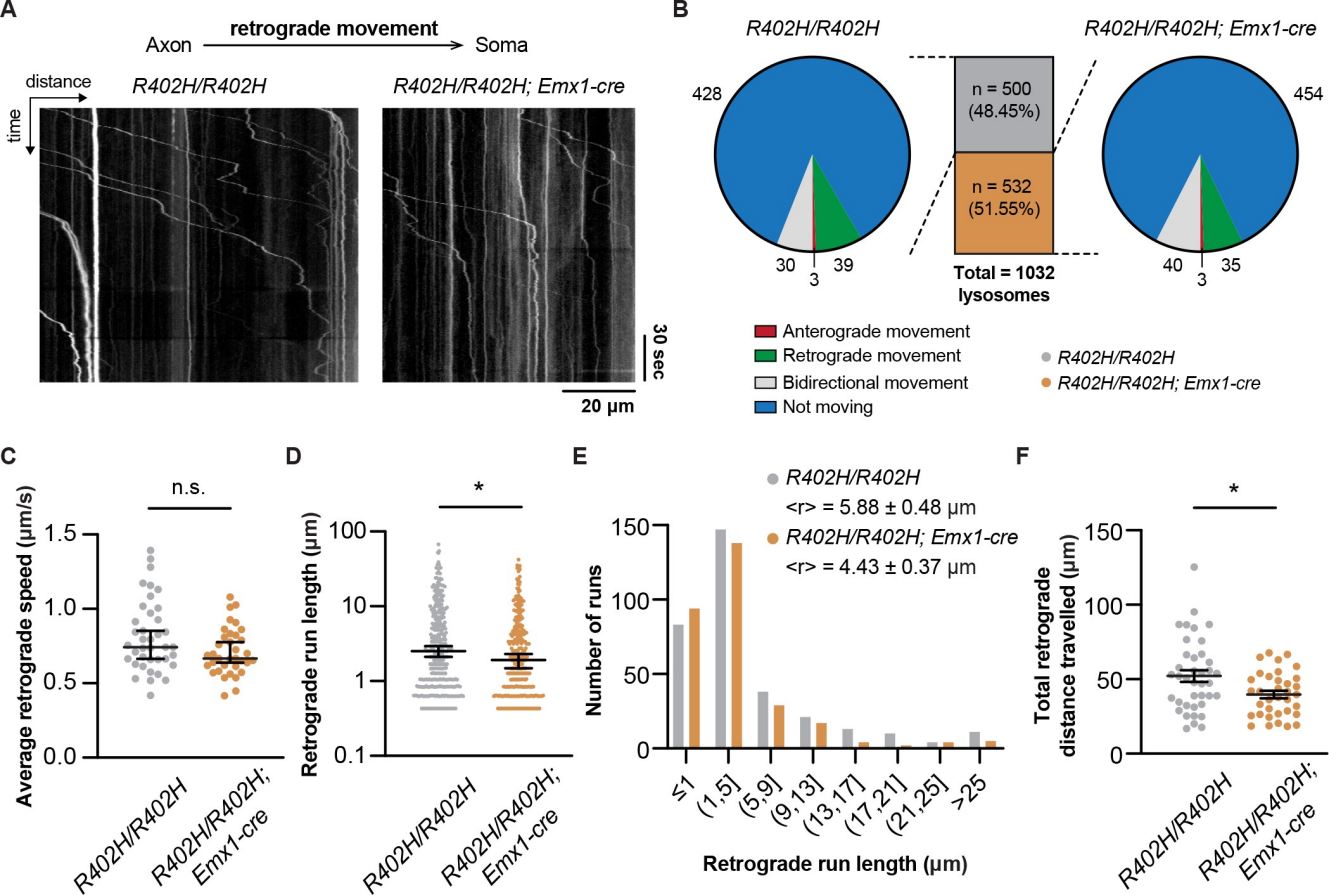
The R402H mutation perturbs dynein-mediated lysosomal transport. (A) Representative kymographs generated from lysosomes tracked in E18.5 primary cortical neurons in *R402H/R402H* controls and *R402H/R402H; Emx1-cre* mutants at DIV3. (B) Lysosomes (n = 1032) were classified according to their movement: anterograde, retrograde, bidirectional, immobile. There was no significant difference in the types of movement when comparing genotypes (Chi-square: 3.078, DF = 3, P = 0.3797). (C) We observed no difference in the average retrograde speed when comparing genotypes (n = 39 lysosomes for *R402H/R402H* controls and n = 35 lysosomes for *R402H/R402H; Emx1-cre* mutants P>0.05), but a significant reduction in lysosomal retrograde run length (D) in *R402H/R402H; Emx1-cre* mutants (n = 343 runs for *R402H/R402H* controls and n = 309 runs for *R402H/R402H; Emx1-cre* mutants P<0.05, with median retrograde run length of 2.52 μm for *R402H/R402H* and 1.91 μm for *R402H/R402H; Emx1-cre* animals). (E) Distribution of retrograde run lengths for *R402H/R402H* controls (in grey) and *R402H/R402H; Emx1-cre* mutants (in orange). (F) Total distance travelled in the retrograde direction is significantly reduced in *R402H/R402H; Emx1-cre* mutants. (n = 39 lysosomes for *R402H/R402H* controls and n = 35 lysosomes for *R402H/R402H; Emx1-cre* mutants P<0.05). *P<0.05. Error bars in C and D show the median ± 95% CI, and in F the mean ± s.e.m.

**Fig 8:**
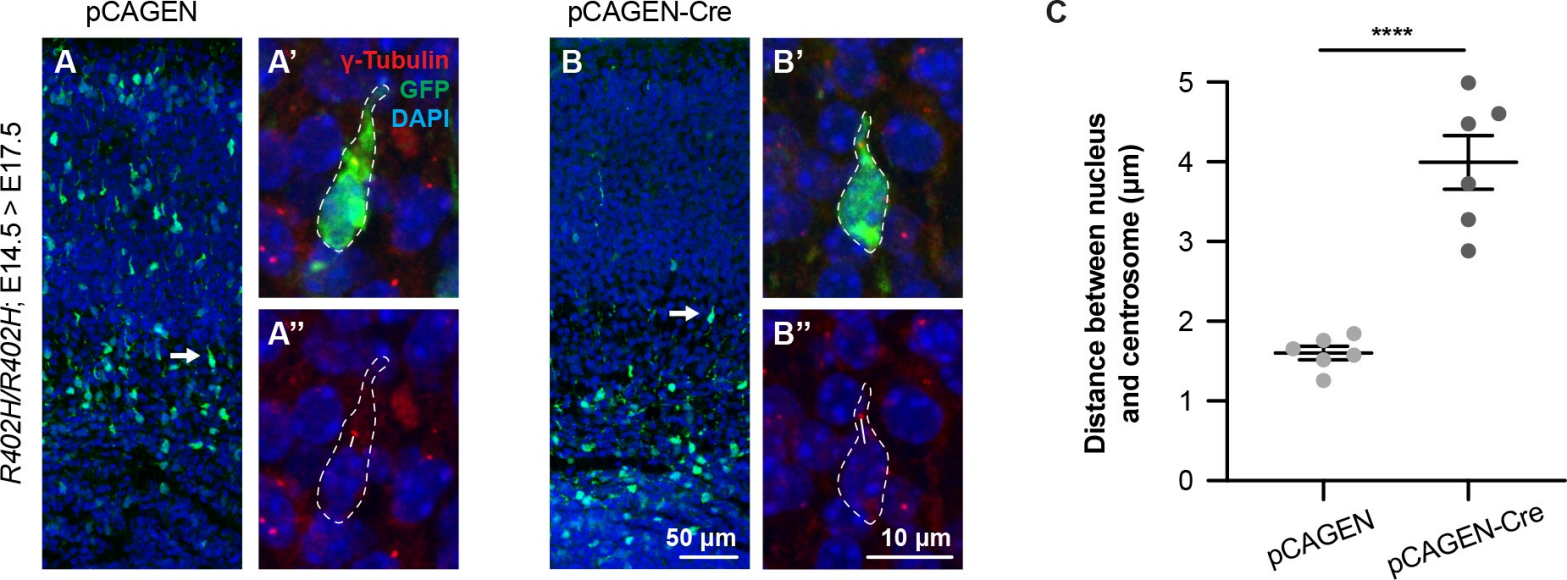
Acute expression of the R402H mutation perturbs nucleus-centrosome coupling in migrating neurons. (A-B) *R402H/R402H* animals were electroporated at E14.5 with either a pCAGEN control vector or pCAGEN-Cre vector to induce expression of the R402H mutation. Embryos were collected at E17.5 and sections stained with antibodies against GFP to label electroporated neurons and γ-Tubulin to identify the centrosomes. A’, A” and B’, B” show high magnification images of migrating neurons (arrow). (C) Quantification reveals a significant increase in the distance between the nucleus and centrosome in migrating neurons expressing the R402H allele in comparison to controls (n = 6 animals, minimum 6 cells per animal, P<0.0001). Error bars in C show the mean ± s.e.m. ****P< 0.0001. Scale bars show 50 μm in B and 10 μm in B”.

Given the results of our proteomic analysis we asked whether dynein-mediated lysosomal transport is perturbed in *R402H/R402H*; *Emx1-cre* animals. To assess this, we cultured primary cortical neurons from *R402H/R402H*; *Emx1-cre* mutants and littermate controls aged E18.5 and performed live cell imaging using Lysotracker to label lysosomes (S1 and S2 Videos) [32]. We generated kymographs and classified each lysosome according to its movement (Fig 7A and S7 Fig). The proportion of anterograde, retrograde, bidirectional and non-moving lysosomes was similar in mutants and controls (Fig 7B) (n = 500 lysosomes for *R402H/R402H* and n = 532 lysosomes for *R402H/R402H; Emx1-cre* Chi-square: 3.078, DF = 3, P = 0.3797). We focused on the lysosomes moving in the retrograde direction, quantitating the speed, run length and total distance (Fig 7C–7F). We found no significant difference in the average speed of lysosomes when comparing *R402H/R402H*; *Emx1-cre* mutants and littermate controls; however, but did observe a significant reduction in run length (Fig 7C and 7D) (n = 343 runs for *R402H/R402H* and n = 309 runs for *R402H/R402H; Emx1-cre* P<0.05). When representing retrograde runs as a frequency distribution, we found a slight enrichment of shorter runs in *R402H/R402H*; *Emx1-cre* mutants (Fig 7E). This result correlated with the total distance travelled by lysosomes towards the soma, which was significantly reduced in *R402H/R402H; Emx1-cre* mutants in comparison to littermate controls (Fig 7F) (n = 39 lysosomes for *R402H/R402H* and n = 35 lysosomes for *R402H/R402H; Emx1-cre* P<0.05). The dynein complex is known to play a critical role in neuronal migration as it generates the force necessary to move the nucleus towards the microtubule organising center [5,33]. Previous studies have shown that dynein perturbation disrupts nucleus-centrosome coupling causing an impairment in nuclear translocation [5,34]. In light of these data we analysed nucleus-centrosome coupling in our *R402H/R402H* mice following *in utero* electroporation of a pCAGEN*–Cre* vector at E14.5, and harvesting at E17.5. We labelled the centrosome with gamma-tubulin and measured the distance from the nucleus to the centrosome in neurons entering the cortical plate (Fig 8A–8C). This revealed a statistically significant increase in nucleus-centrosomal distance when the R402H allele is expressed in comparison to controls (n = 6, *R402H/R402H;* pCAGEN vs. *R402H/R402H;* pCAGEN-Cre, P<0.0001). Taken together these results show that the R402H mutation perturbs dynein-mediated transport and coupling of the nucleus to the microtubule organising center.

## Discussion

In this study we have investigated the pathology and molecular mechanisms that result from an R402H mutation in TUBA1A, a variant that is known to cause lissencephaly in the human population. To do so we generated a R402H conditional mouse model and have shown that it recapitulates multiple aspects of the human disease phenotype. Heterozygous animals present with laminar defects in the cortex and hippocampus, as well as cerebellar and striatal hypoplasia. We attribute the laminar abnormalities observed in the forebrain to defects in neuronal migration, which is exacerbated in homozygous animals. In homozygotes the developing cortical plate is inverted and there is a significant increase in neuronal cell death which is associated with postnatal lethality. Exploiting *in utero* electroporation, we have shown that acute expression of the R402H allele perturbs neuronal migration in a cell-autonomous manner, decoupling the nucleus from the centrosome. While there are a number of existing mouse mutants for TUBA1A, this animal is the first to directly recapitulate a known disease-causing variant. Consistent with the data presented in this paper, the *Jenna* (S140G), *Tuba1a*^ND^ (N102D), and the *Tuba1a*^*M101736*^ (D47G) mouse mutants all present with neuroanatomical phenotypes indicative of impairments in neuronal migration and/or cell death [20,35,36]. Interestingly, the ablation of TUBA1A by CRISPR-mediated genome editing (*Tuba1a*^*d4304/d4304*^, *Tuba1a*^*d4262/d4262*^) results in viable heterozygous animals, that are not reported to have laminar abnormalities in the forebrain [37]. In contrast the homozygous ablation of *Tuba1a* results in lethality, with severe brain malformations with enlarged ventricles [37]. These data, coupled with the results presented in this manuscript indicate that TUBA1A is necessary for cortical development in vertebrates and that the R402H variant acts as a gain of function. This mode of action is supported by the work of Aiken and colleagues who have convincingly shown that ectopic overexpression of the R402H mutation in the developing murine cortex disrupts neuronal migration [25].

To explore the molecular mechanisms that are perturbed by the R402H mutation we sedimented microtubules from the forebrain of *R402H/R402H* animals and performed quantitative mass spectrometry. This resulted in the identification of 286 proteins that were significantly dysregulated in *R402H/R402H* mutants, surprisingly only 7 of these were reported to be MAPs (VAPA, VAPB, REEP1, EZRIN, PRNP, KIF5C and DYNC1l1). While a proportion of the remaining 279 proteins may be uncharacterised MAPs, it is probable that some form complexes with MAPs. For instance, Hap1 is known to interact with dynein participating in vesicular trafficking [38] and β-catenin immunoprecipitates with ezrin [39]. An alternative possibility is that the pellet analysed included precipitated proteins that have simply co-sedimented with the microtubules during sample preparation [40]. Previous work by Yu and colleagues developed a system to identify tubulin associated proteins (TAPs), by expressing a TUBB3-P2A-bio-Sumo-GFP-TUBA1A construct in HEK293T cells. Tubulin heterodimers were then purified using a biotinylated tag and mass spectroscopy performed, resulting in the identification of 3091 putative TAPs. In our study we identified a total of 5283 putative MAPs of which 933 overlap with the Yu dataset. The larger number of proteins identified in our study may be attributed to the fact that we assessed microtubule bound proteins (as opposed to TAPs), our analysis was performed in an *in vivo* setting (as opposed to HEK293T cells), and because we relied on sedimentation (as opposed to tagged proteins).

Western blot analysis confirmed that 5 out of 7 MAPs (VAPB, REEP1, EZRIN, PRNP and DYNC1l1/2) identified by mass spectrometry were dysregulated in *R402H/R402H*, *Emx1-cre* animals. We also observed a reduction in the levels of VAPA in mutant animals in lysates (P = 0.15), but this reduction was not statistically significant. Our inability to confirm a reduction of microtubule associated KIF5C in *R402H/R402H*, *Emx1-cre* animals may be due to the reduced sensitivity of Western blot analysis in comparison to spectroscopic methods or it may be a false positive. VAPA and VAPB are vesicle-associated membrane proteins that are associated with axonal transport of mitochondria and in the case of VAPB implicated in the pathology of amyotrophic lateral sclerosis (*ALS8*) [41,42]. REEP1 plays an important role in the organisation of the microtubule endoplasmic reticulum network, is expressed in the developing cortex and is known to cause spastic paraplegia 31 [43,44]. EZRIN is a cytoskeletal linker that is associated with the plasma membrane and has been shown to regulate RhoA/Rho kinase; it affects neuritogenesis and is highly expressed in migrating neurons within the intermediate zone [45,46]. PRNP encodes the prion protein PrP^C^, responsible for the neurodegenerative process in Creutzfeldt-Jakob disease and has been shown to influence the stability and assembly of microtubules [47,48]. Interestingly, it has been shown that retrograde transport of PrP^C^ within vesicles in hippocampal neurons is mediated by the dynein motor [49]. In the case of VAPB, REEP1, EZRIN and PRNP, we unexpectedly found that the R402H mutation influences the total level of proteins in forebrain lysates. This reduction could be a consequence of a negative feedback loop whereby a reduction in microtubule binding due to the R402H mutation influences the levels of these proteins, but not their transcript.

Our finding that dynein binding and functionality is perturbed by the R402H mutation mirrors the conclusions reached by Aiken and colleagues, who mutated the equivalent tubulin residue (R403) in yeast. They showed that GFP-*tub1*-R403H still incorporates into microtubules and that dynein is correctly recruited to the plus-end of microtubules, but reported that dynein mediated microtubule sliding events during mitosis were impaired [25]. By deleting the minor tubulin *Tub3*, they further demonstrated that this phenotype scales with the ratio of the R403H allele present in cells. This result is consistent with our observation that a more severe neuronal migration phenotype occurs in homozygous *R402H/R402H* animals compared to heterozygous *R402H/+* animals. We have further demonstrated that *in vivo* expression of the R402H mutation decouples the nucleus from the centrosome, consistent with a defect in dynein-mediated nucleokinesis. Interestingly, patients with dynein mutations (*DYNC1H1*) present with a range of developmental phenotypes including polymicrogyria, dysgenesis of the corpus callosum and dysmorphic basal ganglia [7,50]. Dynein mutations are not, however, limited to patients with developmental phenotypes. They have also been reported in individuals with degenerative disorders such as Charcot–Marie–Tooth disease (CMT2) and spinal muscular atrophy [51,52]. It is therefore conceivable that dynein dysfunction contributes to both the migratory and cell death phenotype that we observe in *R402H/R402H* animals.

In summation, our data are consistent with a model whereby the R402H variant is able to fold and incorporate into microtubules, but acts as gain of function by perturbing the binding and/or protein levels of multiple MAPs. We have shown that this is not limited to dynein alone, but includes other MAPs including VAPB, REEP1, EZRIN and PRNP. Future studies will explore what role these proteins play in neuronal migration and/or survival, providing a foundation to design appropriate therapeutic interventions.

## Materials and methods

### Ethics statement

All experiments were carried out according to legal requirements and covered by an approved license (M58/006093/2011/14) from the City of Vienna.

### Animal generation, genotyping and maintenance

Mice carrying the *Tuba1a* R402H allele were generated by Ozgene (Perth, Australia) exploiting their go Germline platform. Briefly, targeting constructs were electroporated into C57/BL6 ES cells and positive clones selected by southern blot analysis following digestion with Nde I. Positive clones were injected into blastocysts to generate chimeras, which were backcrossed to C57/BL6 animals. Animals were genotyped by PCR amplification of *Tuba1a* locus using primers: ATGCTGCCAATAACTATGCT and TGGGAGAAAAGGAAATGTAA. Transgenic mice expressing Cre recombinase were genotyped using the primers: GCTCGACCAGTTTAGTTACCC and TCGCGATTATCTTCTATATCTTCAG. We sequenced both the *Tuba1a* genomic locus and cDNA which confirmed that R402H is the only coding variant present. Animals were housed at the animal research laboratories of the Institute of Molecular Pathology on a 14:10 hour light:dark cycle. Food and water were provided *ad libitum*.

### Immunohistochemistry

E18.5 embryos and P0, P6 and P10 brains were drop-fixed in 4% paraformaldehyde, fixed overnight, and then dehydrated in 30% sucrose before embedding in Neg-50 Frozen Section Medium (Thermo Fisher Scientific, 6502). Littermate controls were sectioned coronally or sagittally (12 μm thick). Adult animals (8–10 weeks old) were perfused at a constant flow of approximately 6.5 mL/min with 4% paraformaldehyde, postfixed for 6 hours and then dehydrated in 30% sucrose. Adult brains were sectioned (40 μm thick) and the sections stored in antifreeze solution (30% glycerol, 30% ethylene glycol, with phosphate buffer). Prior to immunostaining, matched sections were dried at room temperature before washing in PBS. All antibodies used, except for CUX1 and ER81, required heat-mediated antigen retrieval. A Tris-based buffer (Vector Laboratories, H-3301) was used for this purpose in which slides were heated to 90°C. The slides were allowed to cool for at least 45 min and washed in PBS before incubation with primary antibodies: FOXP2 (abcam, ab16046, 1:300), NEUN (Millipore, MAB377, 1:400 and 1:300 for cerebellum staining), CUX1 (kindly provided by Dr. Meinrad Busslinger (IMP, Vienna), 1:1500), ER81 (kindly provided by Prof. Silvia Arber (Biozentrum, Basel), 1:5000), cCaspase3 (Cell Signaling, 9661S, 1:500), TBR1 (abcam, ab31940, 1:400), BRN2 (Santa Cruz, sc-393324, 1:250) and Calbindin (Sigma, C7354, 1:500). Antibodies were diluted in PBS+0.3%Triton containing 2% donkey serum (abcam, ab7475). Incubation was done at 4°C, overnight in a humidified chamber. On the following day, slides were washed 3 times in PBS for 5 mins before incubation with secondary antibodies (1:500). The antibodies used were: donkey anti-mouse alexa fluor 568 (Invitrogen, A10037), donkey anti-mouse alexa fluor 488 (Invitrogen, A21202), donkey anti-rabbit alexa fluor 568 (Invitrogen, A10042), donkey anti-rabbit alexa fluor 488 (Invitrogen, A21206), donkey anti-rat alexa fluor 488 (Invitrogen, A21208). Sections were counterstained with DAPI (1:1000, Thermo Fisher Scientific H3569) before mounting with fluorescence mounting medium (Dako, S3023). Sections were imaged with a scanning confocal microscope (LSM710 Zeiss), employing a 10x/0.30 EC Plan-Neofluar or a 20x/0.8 Plan-Apochromat objective. A negative control was always included where primary antibodies were omitted. Quantification of cortical layer-markers was performed on four (adult) or five (E18.5) images per animal using ImageJ (NIH, version 2.0.0-rc-69/1.52s). For each antibody, positive cells were manually counted in a 300 μm-wide box placed across the somatosensory cortex and divided in ten equally spaced bins. Cortical thickness in adults was measured in four sections per animal stained with NEUN. The total number of cCaspase3 positive cells was manually counted in a 300 μm-wide box placed over the cortex in five sections per animal. To assess the morphology of the cerebellum folium III was imaged and granule cells quantitated in three sections per animal. To quantitate the distribution over the cerebellar layers, two 150 μm-wide boxes were placed over two regions of folium III. The number of granule cells per layer was manually counted, using ImageJ. All quantifications were performed blind to genotype.

### Nissl staining

Slides were washed three times in PBS before incubation with Nissl stain (0.1% cresyl violet acetate; Sigma, C5042), and dehydrated in increasing concentrations of ethanol (30%, 70%, 95% and 100%). Slides were kept in xylol prior to coversliping with DPX mounting media. The slides were then digitized using a Pannoramic 250 Flash II slide scanner (3DHISTECH), with a 20x/0.8 Plan-Apochromat objective. To quantify the cortical thickness four (E18.5) sections per animal were measured with ImageJ. Quantifications were performed blind to genotype.

### BrdU injection and staining

Pregnant mice were injected intraperitoneally with BrdU (5 μL at 20 mg/mL per gram of mouse body weight) at E14.5. Pups were collected at birth (P0) or at postnatal day 10 (P10) and samples drop-fixed in 4% paraformaldehyde overnight, before dehydration in 30% sucrose and embedding in Neg-50 Frozen Section Medium. Littermate controls were sectioned on a cryostat (12 μm) and stained against BrdU using a permanent staining protocol. Briefly, the sections were pre-treated for 5 min with 3% H_2_O_2_ and 0.1% NaN_3_ in water to block endogenous peroxidases. Heat-mediated antigen retrieval was performed using a tris-based buffer (Vector Laboratories, H-3301), followed by an HCl treatment (2N in water) for 70 min at 37°C. Sections were incubated with the primary antibody (1:300, Bio-Rad, OBT0030) prepared in 2% milk in PBS + 0.3% Triton at 4°C, overnight. The next day, sections were washed and incubated with a species-specific secondary antibody, conjugated to horseradish peroxidase (ImmPRESS HRP anti-rat detection kit, Vector Laboratories, MP-7404) for 1 h at room temperature. 3,3'-diaminobenzidine (DAB, Vector Laboratories SK-4100) was used as substrate for HRP. Finally, sections were dehydrated in increasing concentrations of ethanol before mounting with DPX mounting medium (Sigma-Aldrich, 06522). Matching sections were scanned and four images per animal were quantitated using ImageJ. For P0 and P10 brains, BrdU-positive cells were manually counted in a 370 μm-wide box, placed across the cortex and divided in ten equally spaced bins. The different layers of the hippocampus were manually segmented and the BrdU-positive cells, quantified using ImageJ. All quantifications were done blind to genotype.

### *In utero* electroporation

*In utero* electroporations were performed as previously described [53]. For these experiments, the following plasmids were used: pCAGEN (control) or pCAGEN-Cre (used to induce the expression of the R402H mutant) which were injected at 1 μg/μL, together with pCAGEN-GFP at 0.2 μg/μL to label the migrating neurons. Electroporations were performed on E14.5 pregnant *R402H/R402H* females and the embryos collected at E17.5. Following fixation and dehydration, the samples were sliced (12 μm thick) and stained with a GFP antibody. Image acquisition was done on a laser scanning confocal microscope (LSM710 Zeiss). Five images per animal were analysed and the number of GFP positive cells was counted manually using ImageJ, blind to the condition. To assess the distribution of the labelled cells, we divided the cortex in different zones: ventricular zone, the subventricular zone, the inner, medial, and outer intermediate zone (iIZ, mIZ, oIZ), and the inner, medial, and outer cortical plate (iCP, mCP, oCP) [53]. These were defined based on cell density, using the DAPI channel as a reference. Inner/medial/outer sub-zones were defined by sub-diving each cortical zone in three equally spaced layers. To quantify the distance between the nucleus and centrosome, the sections were stained with antibodies against GFP (abcam, ab13970, 1:1000) and gamma-tubulin (Sigma, T6557, 1:100), and counterstained with DAPI. Ten to twelve images were acquired on a laser scanning confocal microscope (LSM800 Zeiss) equipped with a 20x/0.8 Pan-Apochromat DIC objective. Z-stacks were collected at 1 μm steps and maximum projection images generated before quantification. The distance between the nucleus and centrosome was measured manually in ImageJ, blinded to the genotype, in GFP positive migrating neurons in the intermediate zone, with a clear bipolar morphology and identifiable centrosome within the leading process.

### Microtubule sedimentation assay

Microtubules pellets were prepared as previously described [7]. Briefly, forebrain samples were isolated from E18.5 embryos by dissection in cold PBS and subsequent snap-frozen in liquid nitrogen. Protein lysates of wild-type and mutant embryos were prepared simultaneously in tubulin buffer: 100 mM PIPES pH 6.9 (Sigma, P6757), 1 mM MgCl_2_ and 1 mM EGTA (Sigma, E3889) supplemented with protease inhibitor (Roche, 04693159001). The tissue was homogenized by pipetting the cell suspension, kept on ice. After incubation for 15 min on ice, the lysate was centrifuged at maximum speed at 4°C in a tabletop centrifuge, followed by two ultracentrifugations at 60 000 rpm at 4°C for 15 min. Total protein concentration was quantified using Bradford reagent (Bio-Rad, 5000006) and 70% of the cleared lysate was used in the polymerization reaction (approximately 60 μl). For microtubule polymerisation taxol (Sigma, T7191), GTP (Sigma, G8877) and AMP-PNP (Sigma, 10102547001) were added to the lysate to a final concentration of 20 μM, 1 mM and 2 mM, respectively. Microtubule polymerization was performed at 37°C for 30 min. To pellet taxol-stabilized microtubules and microtubule-associated proteins, the reaction was loaded onto a sucrose cushion prepared with 1 M sucrose, 10 μM taxol, 0.5 mM GTP and 1 mM AMPPNP. The samples were then centrifuged at 60 000 rpm at 37°C for 20 min. All ultracentrifugations were performed in a Beckman TLA 100.3 rotor in Optima MAX-XP ultracentrifuge. After removal of the supernatant, the pellet was re-suspended directly in loading dye, boiled at 95°C for 3 min and kept at -20°C. For mass spectrometry analysis the pellets were snap-frozen in liquid nitrogen until further processing.

### Mass spectrometry analysis

Protein pellets were processed using previously described protocols with minor modifications [54]. Briefly, pellets were dissolved in 30 μL of SDT buffer (4% SDS, 0.1 M DTT in 0.1 M TEAB; pH = 7.5) and incubated 10 min at 95°C. Protein extracts were mixed with 200 μL of UA solution (8 M urea in 0.1 M TEAB; pH = 8.5), placed into a microcon unit (MWCO = 30 kDa) and centrifuged for 20 min at 14000 x g. All centrifugations were carried out at room temperature unless stated otherwise. After the washing step, proteins were alkylated by adding 100 μL of 0.1 M iodoacetamide in UA with following 20 min incubation in dark and at room temperature. Following centrifugation proteins were digested directly in the ultrafiltration unit by adding 50 μL of TEAB buffer which contains a specific protease trypsin (Mass spectrometry grade; Promega) in 1:40 ratio (protease:protein; w/w). After overnight digestion at 37°C peptides were eluted into a fresh collection tube by centrifugation at 14000 x g. The ultrafiltration unit was then washed twice by adding 50 μL of 50 mM TEAB buffer, centrifuged again, and the eluate collected. Collected tryptic peptides were subsequently labelled with tandem mass tags (TMT). TMT10-plex labelling reagents were equilibrated to room temperature and 41 μL of anhydrous acetonitrile were added. Reagents and the peptide samples (80 μg for each sample) were mixed together and incubated for 1h. Different amounts of the channels were added to correct the median value to 1:1:1:1:1:1:1:1:1:1. Following labelling, peptides were desalted using C18 cartridges (Sep-Pak Vac 1cc-50 mg, Waters). These were equilibrated, then the peptide sample was loaded on the columns without applying pressure. The cartridge was washed 3 times and to elute the peptides, the cartridge was flushed 3 times with 150 μL of 70% ACN and 0.1% FA. The sample was diluted to a volume of 200 μL with 0.1% FA, snap frozen with liquid nitrogen and lyophilized overnight. The lyophilized peptide sample was dissolved in SCX buffer A (5 mM phosphate buffer pH 2.7, 15% ACN). The SCX fractionation was performed on an Ultimate system (Thermo Fisher Scientific) using a TSKgel SP-25W (ToSOH) column (5 μm particles, 1 mm i.d. x 300 mm) at a flow rate of 40 μL/min. For the separation, a ternary gradient was used, starting with 100% buffer A for 10 min, followed by a linear increase to 10% buffer B (5 mM phosphate buffer pH 2.7, 1 M NaCl, 15% ACN) and 50% buffer C (5 mM phosphate buffer pH 6.0, 15% ACN) in 10 min, to 25% buffer B and 50% buffer C in next 10 min, to 50% buffer B and 50% buffer C in next 5 min and an isocratic elution for further 15 min. The flow-through was collected as a single fraction, along the gradient fractions that were collected every minute and stored before analysis. The system used was an UltiMate 3000 RSLC nano system coupled to a Q Exactive HF-X mass spectrometer, equipped with an EASY-spray ion source (Thermo Fisher Scientific) and JailBreak 1.0 adaptor insert for a spray emitter (Phoenix S&T, Inc., USA). Peptides were loaded onto a trap column (Thermo Fisher Scientific, PepMap C18, 5 mm × 300 μm ID, 5 μm particles, 100 Å pore size) at a flow rate of 25 μL/min using 0.1% TFA as mobile phase. After 10 min, the trap column was switched in line with the analytical column (Thermo Fisher Scientific, PepMap C18, 500 mm × 75 μm ID, 2 μm, 100 Å). Peptides were eluted using a flow rate of 230 nL/min, and a binary 2h gradient, respectively 165 min. The gradient starts with the mobile phases: 98% A (water/formic acid, 99.9/0.1, v/v) and 2% B (water/acetonitrile/formic acid, 19.92/80/0.08, v/v/v), increases to 35% B over the next 130 min, followed by a gradient in 5 min to 90% B, stays there for 5 min and decreases in 2 min back to the gradient 98% A and 2% B for equilibration at 30°C. The Q Exactive HF-X mass spectrometer was operated in data-dependent mode, using a full scan (m/z range 380–1650, resolution of 120 000, target value 3x10^6^) followed by MS/MS scans (resolution of 45000, target value 1x10^5^, maximum injection time 250ms) of the 10 most abundant ions. MS/MS spectra were acquired using normalized collision energy 35% and an isolation width of 0.7 m/z. For the detection of the TMT reporter ions a fixed first mass of 110 m/z was set for the MS/MS scans. Precursor ions selected for fragmentation were put on a dynamic exclusion list for 60 s. The intensity threshold was set to 4x10^4^. Raw files were processed with Proteome Discoverer (version 2.3.0.523, Thermo Fisher Scientific, Germany). Database searches were performed using MS Amanda (version 2.0.0.9849) against the SwissProt mouse database (version 2019.01.26). The raw files were loaded as fractions into the processing workflow. Trypsin was defined as the proteolytic enzyme, cleaving after lysine or arginine, except when followed by proline, and up to two missed cleavages were allowed. Precursor and fragment ion tolerance were set to 5 ppm and 15 ppm respectively. Identified spectra were rescored using Percolator and filtered to 1% FDR at the peptide spectrum match level. Protein grouping was performed in Proteome Discoverer applying a strict parsimony principle. Proteins were subsequently filtered to a false discovery rate of 1% at protein level. TMT Reporter ion S/N values were extracted from the most confident centroid mass within an integration tolerance of 20 ppm. Protein quantification was determined based on unique and razor peptides. Statistical significance of differentially abundant peptide/proteins between different conditions was determined using a paired LIMMA test [55]. The functional enrichment analysis was performed using by g:Profiler (version e98_eg45_p14_bca6d38) with g:SCS multiple testing correction method applying significance threshold of 0.05 as previously described [56].

### Western-blotting

Following mass spectrometry analysis of wild-type and mutant derived microtubule pellets, candidate validation was performed by western-blotting. Microtubule pellets were prepared from E18.5 homozygotes and wild-type littermates as described. The lysate was diluted in tubulin buffer to 4 mg/mL and 10 μg loaded per gel. Microtubule pellets were re-suspended in 60 μL of loading dye and 5 μL loaded per gel. Samples were loaded on 4–12% bis-tris pre-cast gels (Thermo Fisher Scientific, NW04125BOX) and transferred onto a nitrocellulose membrane (GE Healthcare, 10600002) via wet-transfer. Blocking was performed using 5% milk prepared in TBS + 0.01% Tween for 1 h at room temperature. Primary antibodies were diluted in blocking solution at the following concentrations: 1:3000 DYNC1I1/2 (Santa Cruz, sc-13524), 1:2000 KIF5C (abcam, ab5630), 1:200 VAPA (Sigma, HPA009174), 1:1000 VAPB (Sigma, HPA013144), 1:1000 REEP1 (Santa Cruz, sc-393242), 1:300 EZRIN (Cell Signaling, 3145), 1:1000 PRNP (abcam, ab52604), 1:200 000 DM1A (α-tubulin, Sigma, T6199), 1:4000 GAPDH (Millipore, MAB374). Following overnight incubation with primary antibody (at 4°C), the membrane was washed 4 times for 10 min in TBS-T. Incubation with HRP-conjugated secondary antibodies was done for 1 h at room temperature. The following antibodies were used: anti-mouse (1:5000, abcam, ab6723) and anti-rabbit (1:10000, abcam, ab6721). The signal was detected using an ECL Western Blotting Detection Reagents kit (GE Healthcare, RPN2106 and RPN2235) and images captured with ChemiDoc Touch Imaging system. When required, membranes were stripped using the appropriate buffer (Thermo Fisher Scientific, 46430). Western blots were quantified using ImageJ.

### mRNA extraction, cDNA preparation and qPCR

Cortices from E18.5 embryos (n = 5) were dissected and snap-frozen in liquid nitrogen. Total RNA was extracted, and cDNA synthesized using commercially available kits (RNeasy Mini Kit, 74104, Qiagen, and QuantiTect Reverse Transcription Kit, Qiagen, 205313). To quantify *Vapb*, *Reep1*, *Ezrin* and *Prnp* mRNA levels in *R402H/R402H* and *R402H/R402H; Emx1-cre* samples, we used SYBR green (SsoAdvanced Universal SYBR Green Supermix, 1725272, Biorad) on a Biorad CFX 384 Real Time Cycler. We used the following primers, either intron spanning or designed to bind to exon-exon junctions: mVapb_qPCR_F/TAGATGTGTGTTTGAATTGCCAGC and mVapb_qPCR_R/TGGATGCACTCGTAGGTATGATTT, mReep1_qPCR_F/CAGGCTGGTGGTGCTTATATTTG and mReep1_qPCR_R/TGGACTTCACAGCCTTGTATGAAT, mEzr_qPCR_F/GTCATTAAGCCCATCGACAAGAAG and mEzr_qPCR_R/CAGGATCCGCTTGTTAATTCTCAG, mPrnp_qPCR_F/ATTCTGCCTTCCTAGTGGTACC and mPrnp_qPCR_R/TAGCCAAGGTTCGCCATGATG,. We used three control genes: *Hprt* (Hprt_qPCR_F/GAACCAGGTTATGACCTAGATTTGTT and Hprt_qPCR_R/CAAGTCTTTCAGTCCTGTCCATAAT), *Tfrc* (Tfrc_qPCR_F/TCGCTTATATTGGGCAGACC and Tfrc_qPCR_R/ATCCAGCCTCACGAGGAGT) and *Pgk1* (Pgk1_qPCR_F/AAAGTCAGCCATGTGAGCACT and Pgk1_qPCR_R/ACTTAGGAGCACAGGAACCAAA). All reactions were performed in technical triplicates. Biological replicates (n = 5) were run together in a 384-well plate, set up using an Agilent Bravo LT96 Liquid Handling system. The geometric mean of the Ct values for the three control genes was calculated and used to determine ΔCt (difference to the mean of the Ct values for each candidate gene). Relative mRNA levels were then determined taking into consideration the qPCR primer efficiency as previously described [31].

### Primary neuron culture and live-cell imaging of lysosomes

Cortical primary neurons were prepared from E18.5 embryos as previously described [57]. The meninges were carefully removed before adding trypsin solution (0.05% Trypsin-EDTA, buffered with 1 M HEPES) and incubating the tissue at 37°C for 15 min. The cortices were then washed three times with HBSS (Gibco, 14025050) before being triturated using P1000 and P200 pipette tips. The resulting cell suspension was filtered through a 40 μm filter (Falcon, 352340) before cell counting. For the lysotracker experiments, 2x10^5^ neurons were seeded in poly-L-lysine coated MatTek dishes (MatTek Corporation, P35G-0-14-C). Following 3 days *in vitro* (DIV), the neuronal cultures were incubated with fresh neurobasal media containing lysotracker diluted at 1:2000 for 25–30 min at 37°C (Thermo Fisher Scientific, L12492). The cultures were washed prior to addition of imaging media (neurobasal medium without phenol red, Thermo Scientific 12348017). Control and homozygous embryos were imaged together and blinded to genotype in four independent experiments. Imaging was performed on a spinning disc microscope equipped with a Hamamatsu EMCCD 9100–13 camera from PerkinElmer UltraView VoX, using a 63x/1.15 LD C-Apochromat water objective. Trafficking was recorded in the axon for 3 min every 0.5 sec. Files were exported from Volocity (version 6.3.0, PerkinElmer) and analysed in ImageJ. Lysosomal trafficking was measured in the axon, considered as the longest process that also showed a thicker protuberance on the cell body, resembling an axon hillock.

### Kymograph analysis and lysosome trafficking

To determine speed and run length, kymographs were generated with the KymoToolBox [58] and the motion of individual lysosomes traced using the Simple Neurite Tracer plug-in for ImageJ. For each kymograph paths were created as a user-defined selection, converted to a segmented line and exported as a ROI, before analysis with a Python-based script. Movement that lasted for at least 3 frames (1.5 sec), was considered a run and was followed until the next change of state. Each track was then classified as retrograde, anterograde, bidirectional or paused, and only tracks traced for at least 60 sec in the retrograde direction were considered. All analysis was done blinded to genotype.

### Statistical methods

All statistical analysis was performed in GraphPad Prism software package (v8.0.2). We employed a variety of statistical tests including unpaired t-tests, one- and two-way repeated measures ANOVAs, with Tukey’s or Sidak’s multiple comparisons test (See S1 Table). Datasets were checked for normality by the Shapiro-Wilk test and whenever the data did not follow a normal distribution, nonparametric tests were applied. These included: Mann-Whitney test and Kruskal-Wallis test with Dunn’s multiple comparisons test. When necessary, a Bonferroni correction for multiple comparisons was applied. Samples and animals were not subject to randomization but were assigned to experimental groups based on their genotype.

## Supporting information

S1 Fig Generation and characterisation of *R402H Tuba1a* mice. (A) Schematic representation of the *Tuba1a* targeting construct designed to conditionally express the R402H patient mutation. After removal of the neomycin cassette, the conditional knock-in (cKI) allele consists of two copies of exon 4, one flanked by LoxP sites and the other carrying the R402H mutation. Upon Cre recombination, the wild-type exon is excised and the mutant exon 4 is expressed. (B) Southern blot analysis of four different clones. The expected size of the wild-type allele is 9.1 kb, and 7 kb for the conditional knock-in. A009F and A044F are both heterozygous for the cKI insertion and A004M and A040F are wild-type mice. (C-H) Representative Nissl stained hippocampal (C-E) and cortical sections (F-H) from adult animals. There is no difference between wild-type (*+/+*), heterozygous (*R402H/+*) and homozygous (*R402H/R402H*) animals in the absence of a Cre recombinase, demonstrating that introduction of the LoxP sites into the *Tuba1a* locus has no effect. (I) Quantification of cortical thickness (n = 3, P>0.05). Error bars show mean ± s.e.m. Scale bars show 500 μm in E and 200 μm in H.
(TIF)

S2 Fig Quantification of the total number of BrdU positive cells at P10 and P0. (A-C) There is no significant difference in the total number of BrdU-labelled neurons when comparing *+/+; Emx1-cre* controls, *R402H/+* controls and *R402H/+; Emx1-cre* mutants in the P10 cortex, P0 cortex or the P10 hippocampus (n = 5, P>0.05). Error bars show mean ± s.e.m. Two-way repeated-measures ANOVA with Tukey’s test for multiple comparisons; n.s.–not significant.
(TIF)

S3 Fig Total number of TBR1+ and BRN2+ cells. (A-B) Quantification of the total number of (A) TBR1+ and (B) BRN2+ neurons in E18.5 sections. Labeled cells were manually counted in a 300 μm-wide box placed across the developing somatosensory cortex. For both layer markers there was no significant difference between *+/+; Emx1-cre* controls and *R402H/+; Emx1-cre* heterozygotes (n = 5, P>0.05), however, there was a significant reduction in TBR1+ and BRN2+ cells in *R402H/R402H; Emx1-cre* homozygotes (n = 5, TBR1: *+/+; Emx1-cre* vs. *R402H/R402H; Emx1-cre* P<0.01, *R402H/+; Emx1-cre* vs. *R402H/R402H; Emx1-cre* P<0.01, BRN2: *+/+; Emx1-cre* vs. *R402H/R402H; Emx1-cre* P<0.05). Error bars show mean ± s.e.m. *P<0.05; **P<0.01.
(TIF)

S4 Fig Western-blot analysis of α-tubulin levels and bioinformatics analysis of mass spectrometry data. (A) Western blot analysis of α-tubulin in brain lysates and microtubule pellets, prepared from *R402H/R402H* and *R402H/R402H; Emx1-cre* E18.5 animals. There is no significant difference between the levels of α-tubulin present in brain lysates (normalized to GAPDH) (B) and in microtubule pellets (C) (n = 3, *R402H/R402H* vs. *R402H/R402H; Emx1-cre* P>0.05). (D) Heatmap showing the reproducibility of the mass spectrometry data between biological replicates (n = 4). Mut/Ctrl 1–4 correspond to four littermate pairs of mutants (*R402H/R402H; Emx1-cre* animals) and corresponding controls (*R402H/R402H)*. Each line denotes one of the 286 proteins (FDR 5%) that showed a significant difference in protein levels. Proteins present at increased levels in mutants (log_2_ fold change >0) are shown in red; those with a decrease (log_2_ fold change <0) are shown in blue.
(TIF)

S5 Fig Western-blot validation of microtubule-associated proteins. (A-G) Full images of western blot membranes for (A) DYNC1I1/2, (B) REEP1, (C) KIF5C, (D) VAPA, (E) PRNP, (F) VAPB and (G) EZRIN. Dashed rectangles indicate the boxes included in Fig 6. Please note that GAPDH loading controls are the same for EZRIN and PRNP in (E, G). That is because the same membrane was probed against several proteins with different size targets. Two pre-stained protein ladders (high molecular weight ladder and broad range ladder) were used and are labeled in each gel. For some membranes a longer exposure was required, and is indicated accordingly.
(TIF)

S6 Fig Expression analysis of candidate MAP genes. (A) qPCR results showing the expression of *Vapb*, *Reep1*, *Ezrin* and *Prnp* in cortices of E18.5 *R402H/R402H* and *R402H/R402H; Emx1-cre* mice (n = 5, *R402H/R402H* vs. *R402H/R402H; Emx1-cre* VAPB: P>0.5, REEP1: P>0.5, EZRIN: P>0.1 and PRNP: P>0.1). Error bars show mean ± s.e.m., n.s.–not significant.
(TIF)

S7 Fig Schematic showing lysosome tracking analyses. (A) Schematic showing kymograph generation and subsequent analysis. Kymographs were generated using a plug-in for ImageJ and each lysosome traced individually. The entire analysis was done blinded to genotype, using a Python-based custom-made script allowing for run length and average speed calculation.
(TIF)

S1 Table Summary of all statistical analysis performed in this study. (XLSX)

S2 Table Mass spectrometry results of 5283 proteins detected in microtubule pellets from *R402H/R402H* controls and *R402H/R420H; Emx1-cre* mutants. (XLSX)

S3 Table Mass spectrometry results of 1330 proteins significantly dysregulated in *R402H/R402H; Emx1-cre* mutants (P-value <0.01). (XLSX)

S4 Table Mass spectrometry results of 286 proteins significantly dysregulated in *R402H/R402H; Emx1-cre* mutants, following the application of an FDR of 5%. (XLSX)

S1 Video Live cell imaging of *R402H/R402H* primary cortical neurons incubated with Lysotracker. Lysosome trafficking was recorded for 3 min every 0.5 sec. The playback speed of the video is 20 fps. This relates to the kymograph presented in Fig 7A.
(AVI)

S2 Video Live imaging of *R402H/R402H; Emx1-cre* primary cortical neurons incubated with Lysotracker. Wild-type and mutant animals were processed simultaneously, and imaging was done blinded to genotype. Lysosome trafficking was recorded for 3 min every 0.5 sec. The playback speed of the video is 20 fps. This relates to the kymograph presented in Fig 7A.
(AVI)
