# Supplementary figures and images for: A proteomic survey of microtubule-associated proteins in a R402H TUBA1A mutant mouse
Source: PLoS Genet. 2020 Nov 2;16(11):e1009104. doi: 10.1371/journal.pgen.1009104 (PMC7660477; doi:10.1371/journal.pgen.1009104)

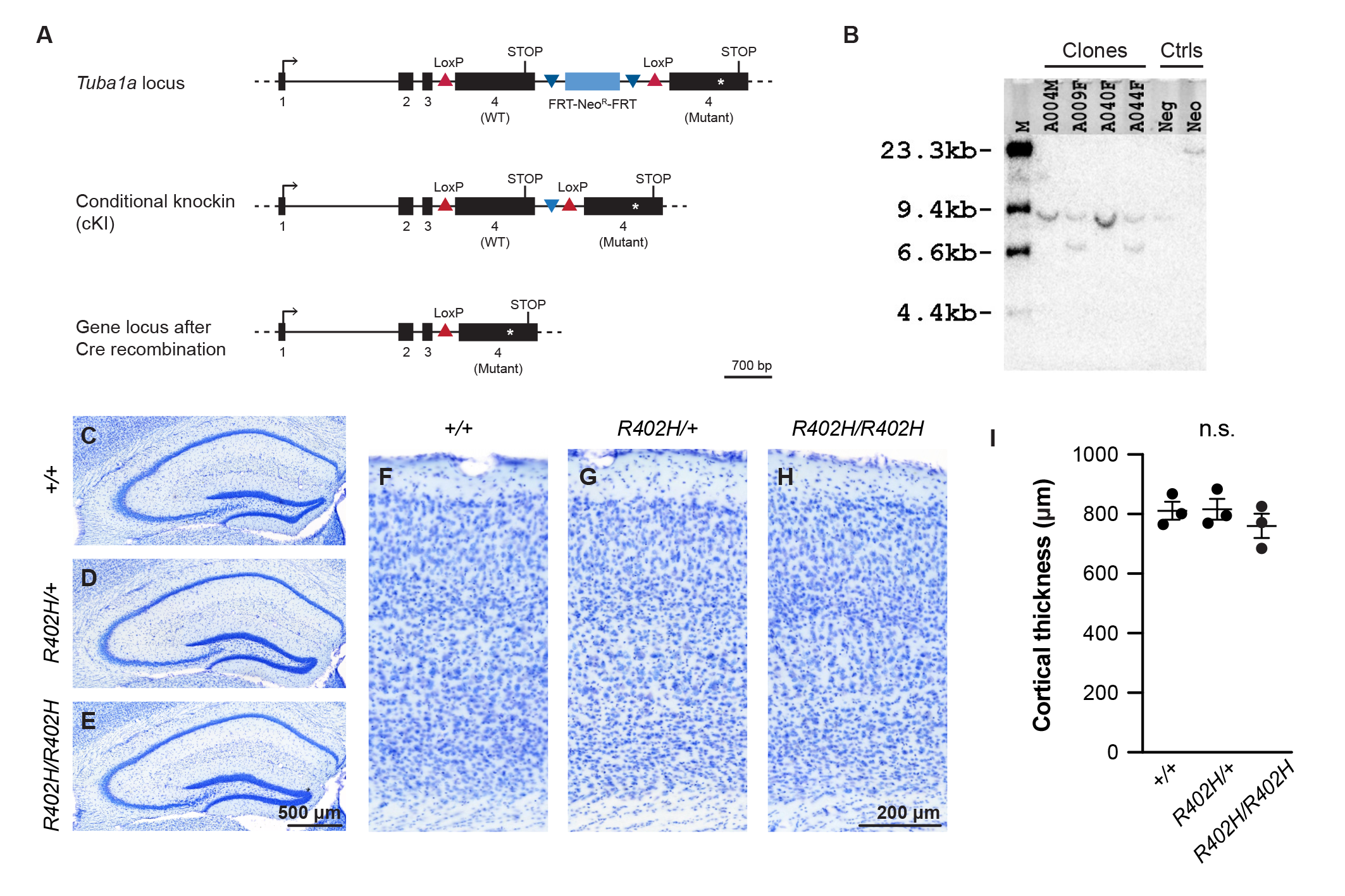

Supplement: S1 Fig — (A) Schematic representation of the Tuba1a targeting construct designed to conditionally express the R402H patient mutation. After removal of the neomycin cassette, the conditional knock-in (cKI) allele consists of two copies of exon 4, one flanked by LoxP sites and the other carrying the R402H mutation. Upon Cre recombination, the wild-type exon is excised and the mutant exon 4 is expressed. (B) Southern blot analysis of four different clones. The expected size of the wild-type allele is 9.1 kb, and 7 kb for the conditional knock-in. A009F and A044F are both heterozygous for the cKI insertion and A004M and A040F are wild-type mice. (C-H) Representative Nissl stained hippocampal (C-E) and cortical sections (F-H) from adult animals. There is no difference between wild-type (+/+), heterozygous (R402H/+) and homozygous (R402H/R402H) animals in the absence of a Cre recombinase, demonstrating that introduction of the LoxP sites into the Tuba1a locus has no effect. (I) Quantification of cortical thickness (n = 3, P>0.05). Error bars show mean ± s.e.m. Scale bars show 500 μm in E and 200 μm in H. (TIF) [file pgen.1009104.s001.tif]

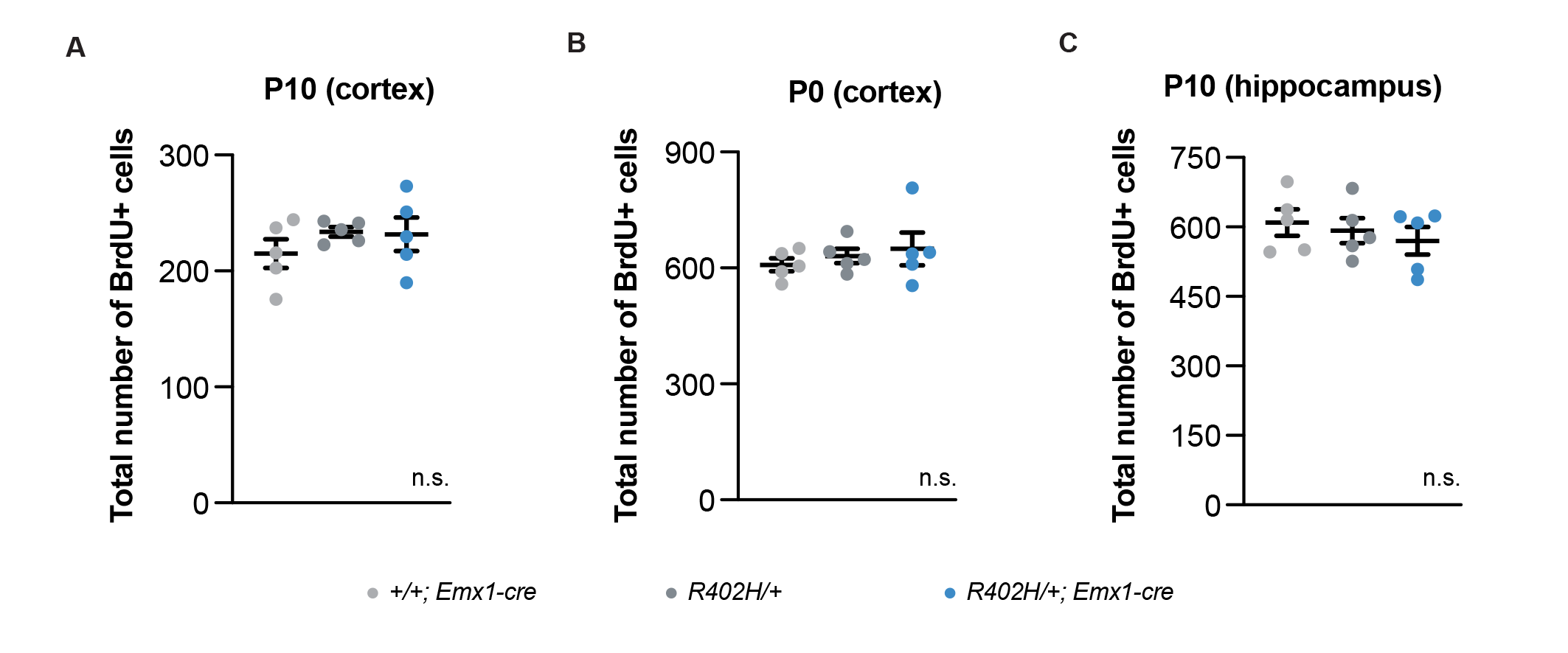

Supplement: S2 Fig — (A-C) There is no significant difference in the total number of BrdU-labelled neurons when comparing +/+; Emx1-cre controls, R402H/+ controls and R402H/+; Emx1-cre mutants in the P10 cortex, P0 cortex or the P10 hippocampus (n = 5, P>0.05). Error bars show mean ± s.e.m. Two-way repeated-measures ANOVA with Tukey’s test for multiple comparisons; n.s.–not significant. (TIF) [file pgen.1009104.s002.tif]

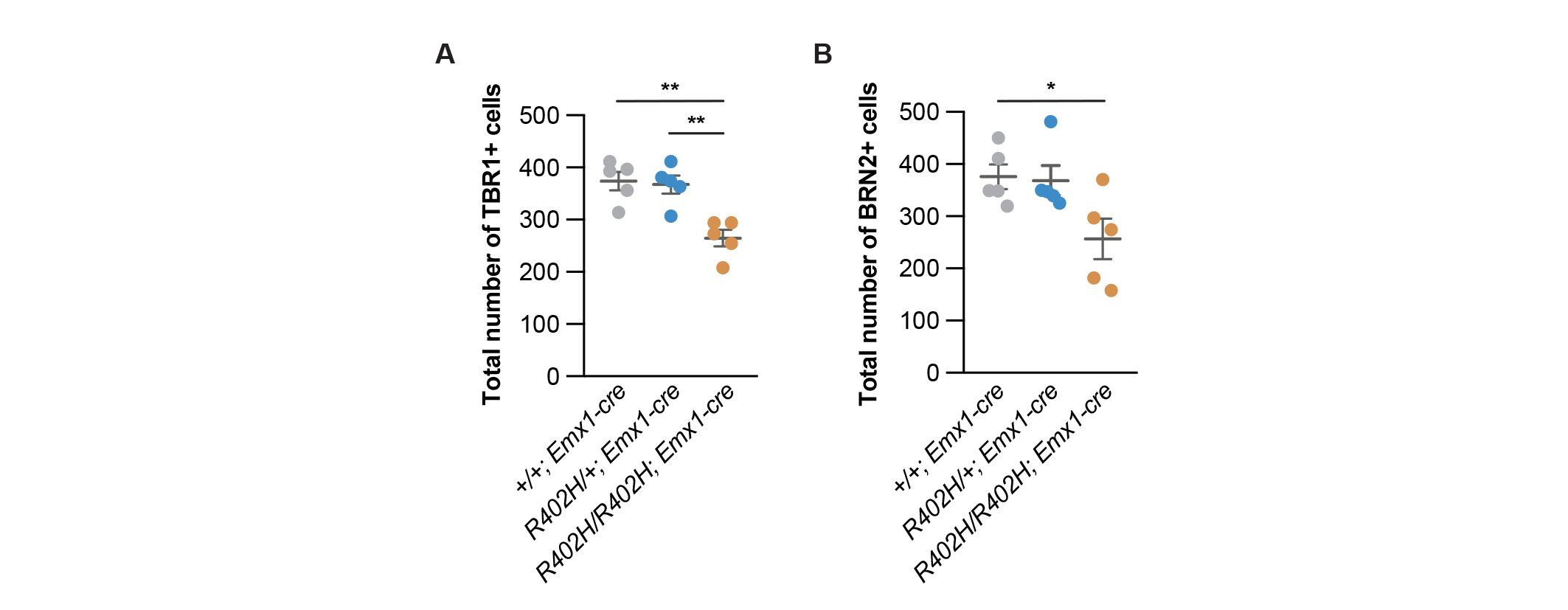

Supplement: S3 Fig — (A-B) Quantification of the total number of (A) TBR1+ and (B) BRN2+ neurons in E18.5 sections. Labeled cells were manually counted in a 300 μm-wide box placed across the developing somatosensory cortex. For both layer markers there was no significant difference between +/+; Emx1-cre controls and R402H/+; Emx1-cre heterozygotes (n = 5, P>0.05), however, there was a significant reduction in TBR1+ and BRN2+ cells in R402H/R402H; Emx1-cre homozygotes (n = 5, TBR1: +/+; Emx1-cre vs. R402H/R402H; Emx1-cre P<0.01, R402H/+; Emx1-cre vs. R402H/R402H; Emx1-cre P<0.01, BRN2: +/+; Emx1-cre vs. R402H/R402H; Emx1-cre P<0.05). Error bars show mean ± s.e.m. *P<0.05; **P<0.01. (TIF) [file pgen.1009104.s003.tif]

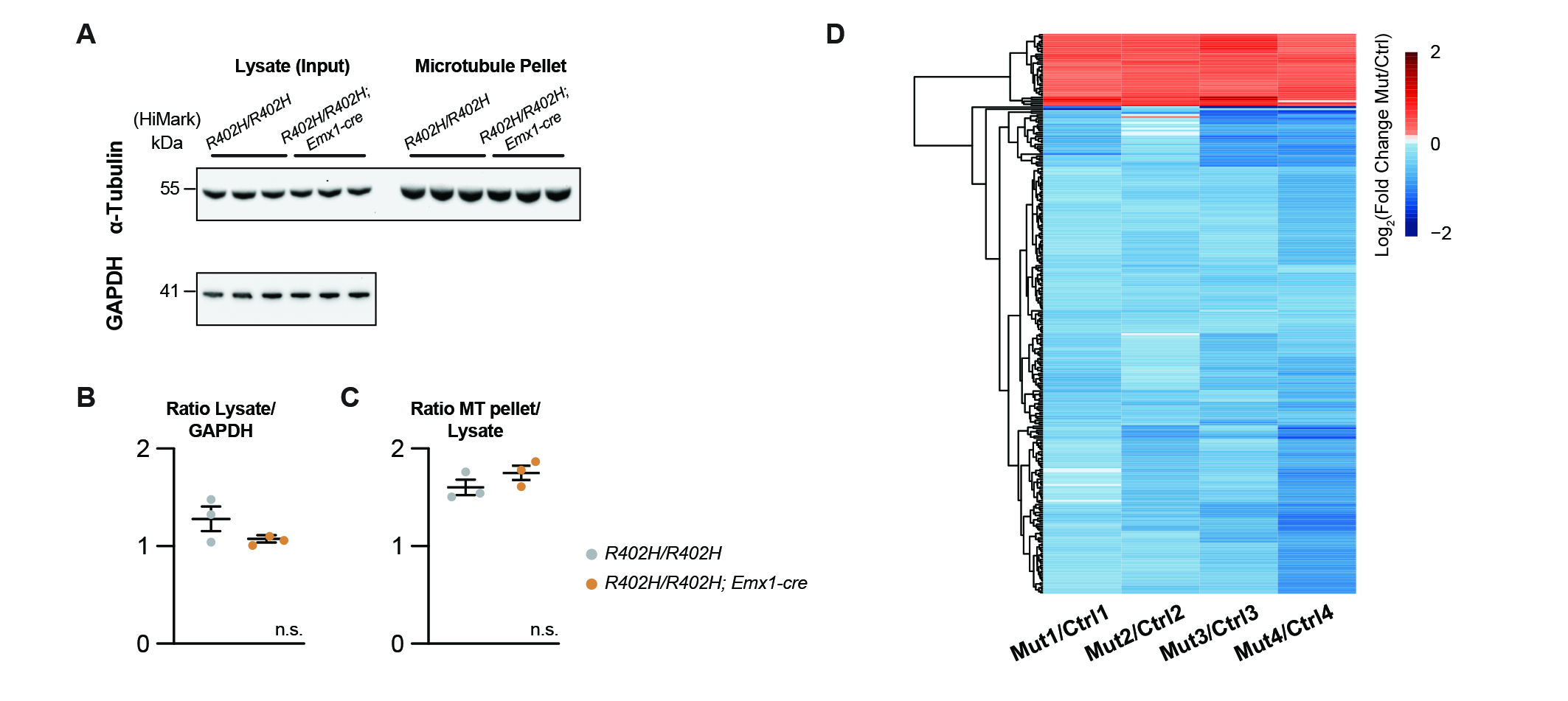

Supplement: S4 Fig — (A) Western blot analysis of α-tubulin in brain lysates and microtubule pellets, prepared from R402H/R402H and R402H/R402H; Emx1-cre E18.5 animals. There is no significant difference between the levels of α-tubulin present in brain lysates (normalized to GAPDH) (B) and in microtubule pellets (C) (n = 3, R402H/R402H vs. R402H/R402H; Emx1-cre P>0.05). (D) Heatmap showing the reproducibility of the mass spectrometry data between biological replicates (n = 4). Mut/Ctrl 1–4 correspond to four littermate pairs of mutants (R402H/R402H; Emx1-cre animals) and corresponding controls (R402H/R402H). Each line denotes one of the 286 proteins (FDR 5%) that showed a significant difference in protein levels. Proteins present at increased levels in mutants (log2 fold change >0) are shown in red; those with a decrease (log2 fold change <0) are shown in blue. (TIF) [file pgen.1009104.s004.tif]

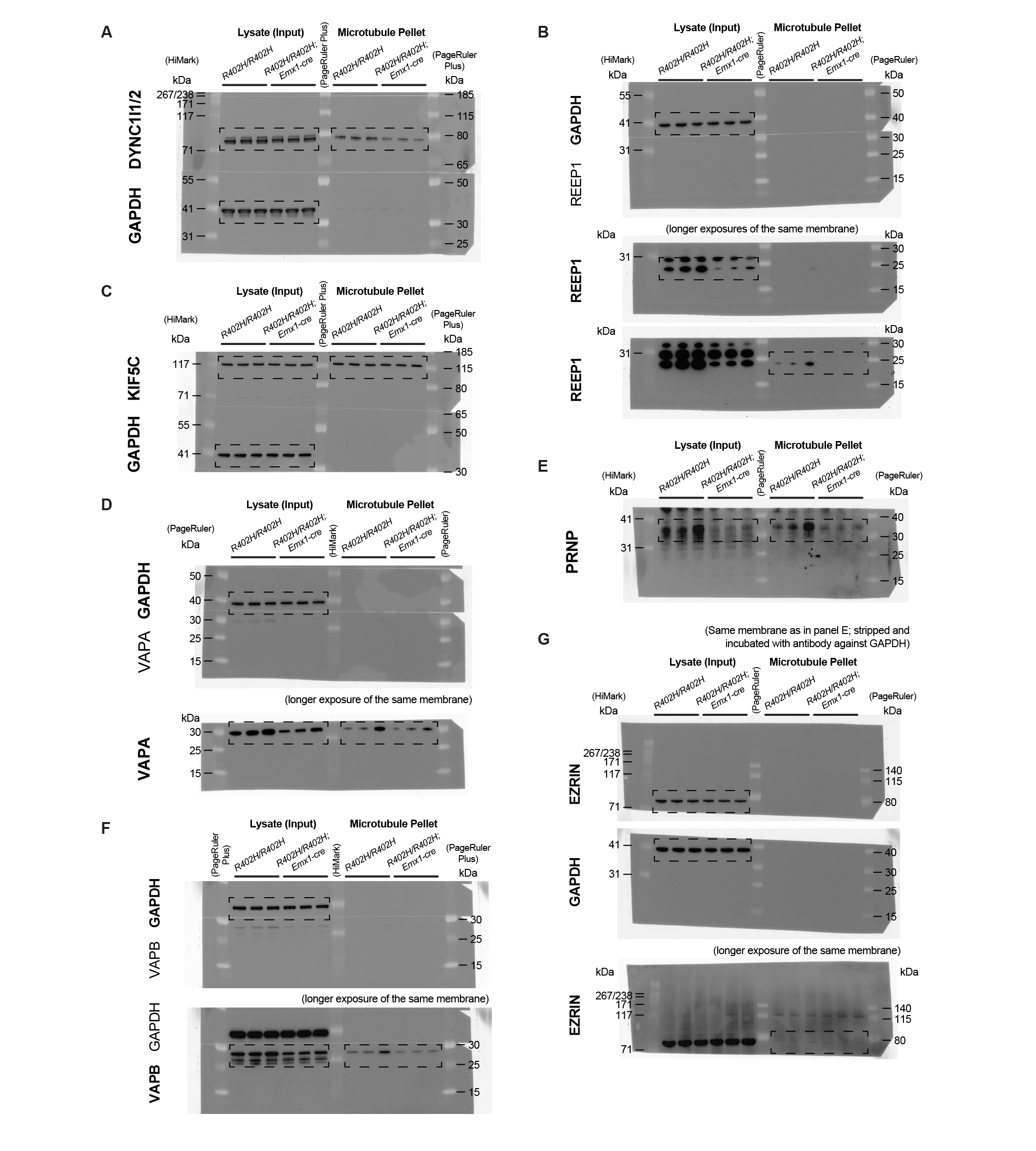

Supplement: S5 Fig — (A-G) Full images of western blot membranes for (A) DYNC1I1/2, (B) REEP1, (C) KIF5C, (D) VAPA, (E) PRNP, (F) VAPB and (G) EZRIN. Dashed rectangles indicate the boxes included in Fig 6. Please note that GAPDH loading controls are the same for EZRIN and PRNP in (E, G). That is because the same membrane was probed against several proteins with different size targets. Two pre-stained protein ladders (high molecular weight ladder and broad range ladder) were used and are labeled in each gel. For some membranes a longer exposure was required, and is indicated accordingly. (TIF) [file pgen.1009104.s005.tif]

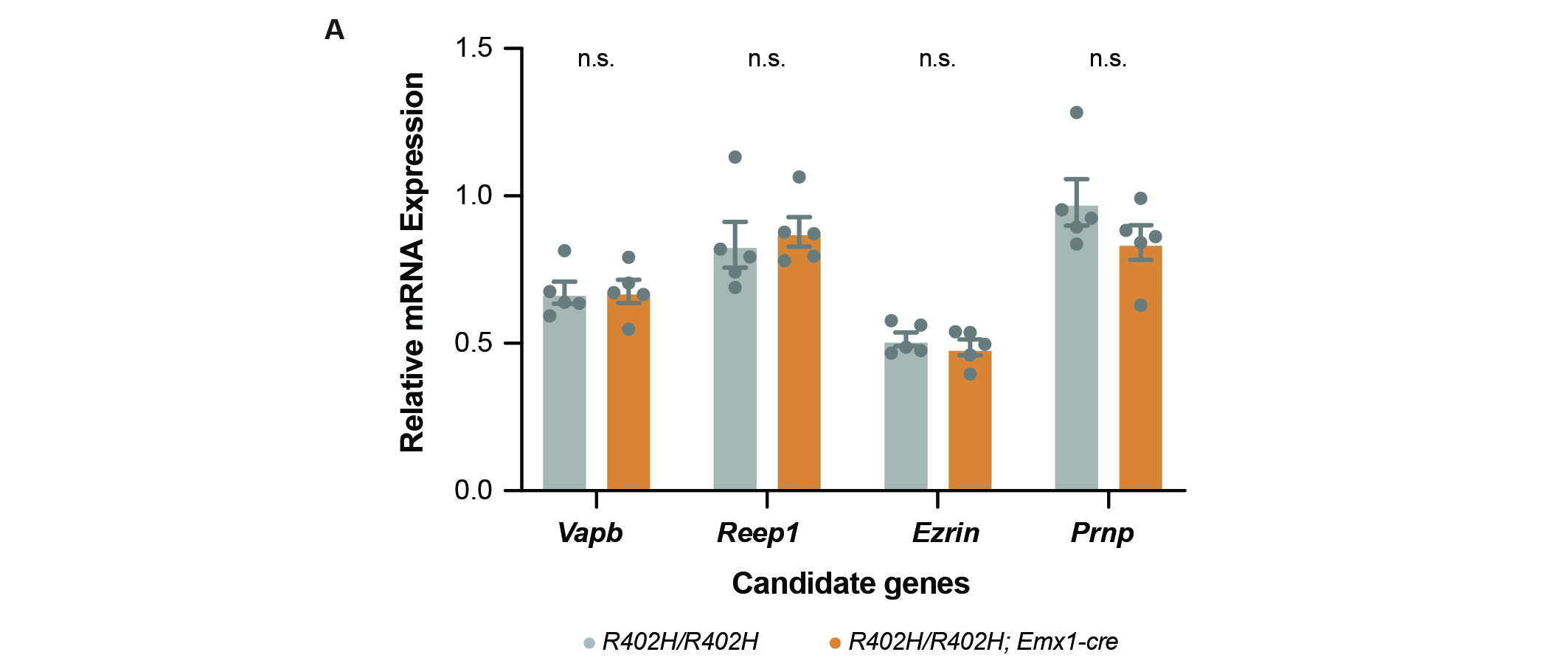

Supplement: S6 Fig — (A) qPCR results showing the expression of Vapb, Reep1, Ezrin and Prnp in cortices of E18.5 R402H/R402H and R402H/R402H; Emx1-cre mice (n = 5, R402H/R402H vs. R402H/R402H; Emx1-cre VAPB: P>0.5, REEP1: P>0.5, EZRIN: P>0.1 and PRNP: P>0.1). Error bars show mean ± s.e.m., n.s.–not significant. (TIF) [file pgen.1009104.s006.tif]

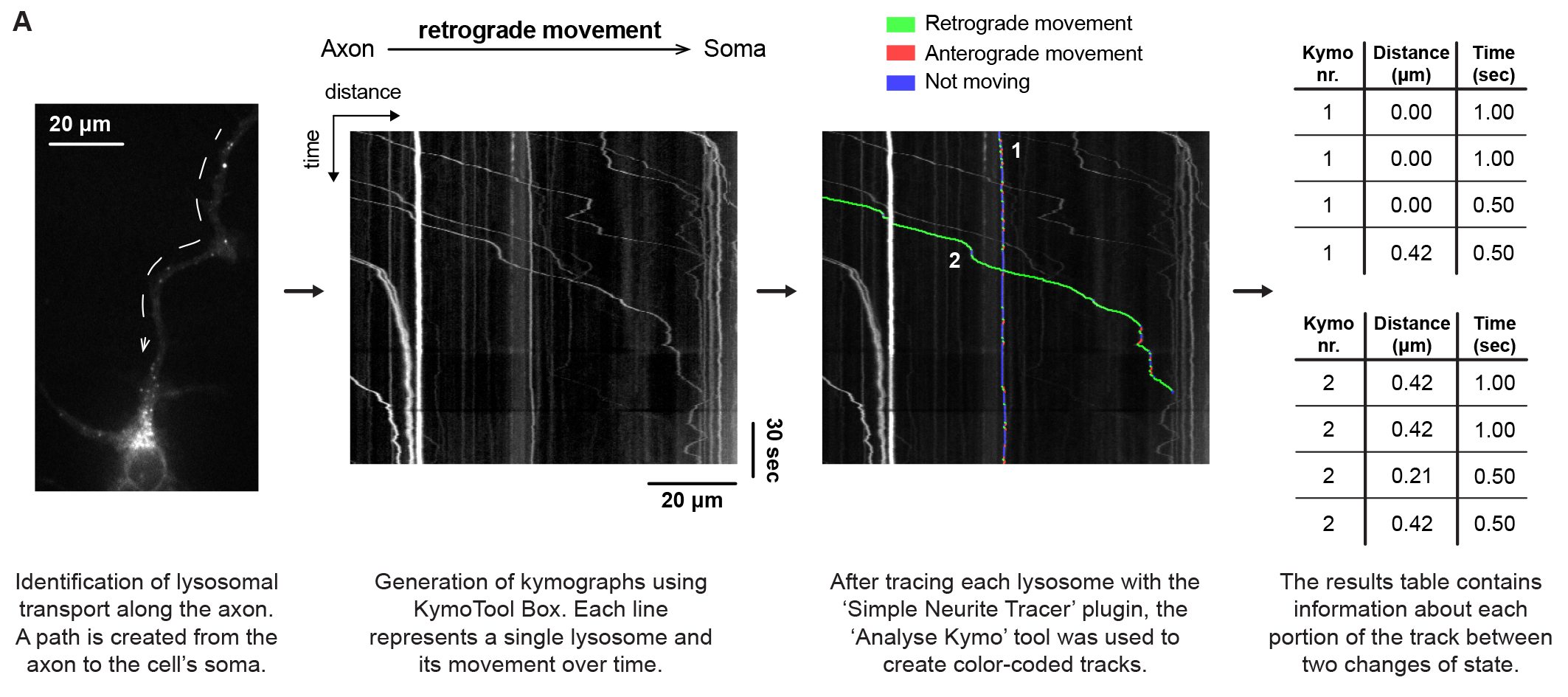

Supplement: S7 Fig — (A) Schematic showing kymograph generation and subsequent analysis. Kymographs were generated using a plug-in for ImageJ and each lysosome traced individually. The entire analysis was done blinded to genotype, using a Python-based custom-made script allowing for run length and average speed calculation. (TIF) [file pgen.1009104.s007.tif]
